# Measuring the refractive index and sub-nanometre surface functionalisation of nanoparticles in suspension†

**DOI:** 10.1039/d2nr00120a

**Published:** 2022-05-05

**Authors:** Niall M. C. Mulkerns, William H. Hoffmann, Javier Ramos-Soriano, Noelia de la Cruz, Teodoro Garcia-Millan, Robert L. Harniman, Ian D. Lindsay, Annela M. Seddon, M. Carmen Galan, Henkjan Gersen

**Affiliations:** H. H. Wills Physics Laboratory, University of Bristol Bristol BS8 1TL UK h.gersen@bristol.ac.uk; Bristol Centre for Functional Nanomaterials, University of Bristol Bristol BS8 1TL UK; School of Chemistry, University of Bristol Bristol BS8 1TS UK

## Abstract

Direct measurements to determine the degree of surface coverage of nanoparticles by functional moieties are rare, with current strategies requiring a high level of expertise and expensive equipment. Here, a practical method to determine the ratio of the volume of the functionalisation layer to the particle volume based on measuring the refractive index of nanoparticles in suspension is proposed. As a proof of concept, this technique is applied to poly(methyl methacrylate) (PMMA) nanoparticles and semicrystalline carbon dots functionalised with different surface moieties, yielding refractive indices that are commensurate to those from previous literature and Mie theory. In doing so, it is demonstrated that this technique is able to optically detect differences in surface functionalisation or composition of nanometre-sized particles. This non-destructive and rapid method is well-suited for *in situ* industrial particle characterisation and biological applications.

## Abstract 2

An optical technique to precisely quantify the amount of active compound associated with nanoparticles in suspension is presented and experimentally verified, enabling differentiation between Angstrom-scale coronas on nanometre-sized carbon dots.

## Graphical abstract

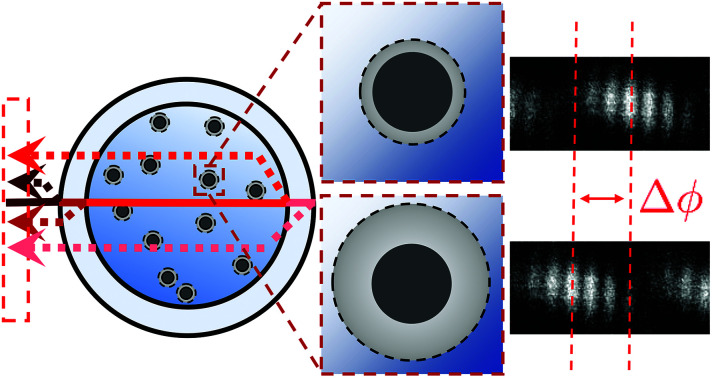

## Introduction

The field of nanotechnology has had a major impact on many research areas such as engineering, electronics, energy, environment, biology, and medicine.^1–3^ There has been an explosion of interest in the development and application of functionalised nanomaterials that can be easily prepared and tailored to specific applications, and as a result of this, the development of robust physico-chemical methods to structurally characterise these materials has become increasingly important.^4–9^

Nanoparticles (NPs) are a ubiquitous and versatile subset of nanomaterials with numerous applications. Carbon dots (CDs), for example, have emerged as a new class of carbon-based fluorescent nanoparticles with a quasi-spherical morphology and unique optical and physico-chemical properties. This is due to their tunable photoluminescence, chemical inertness, high water solubility, low cost of fabrication and very low cytotoxicity.^10–13^ Recent interest has focused on their application as a platform for gene delivery,^14,15^ cell imaging,^16^ and diagnosis through fluorescence.^17,18^ These materials have also found applications in metal sensing,^19^ photo-catalysis,^20^ photosynthesis augmentation,^21^ and photovoltaics.^22^

Many of these potential uses, especially those in biological applications, involve transporting payloads of molecules bound to the outer layer of the nanoparticle in a process known as surface functionalisation. This allows the particles to gain new characteristics and behaviours based on the chemical composition of this corona and its interaction with the surrounding environment, due to the high surface area to volume ratio of nanomaterials. While it is generally assumed that functionalisation of NPs results in a homogeneously coated surface, this is not always the case and very much dependent on the type of payload. Moreover, if the surface functionalisation is non-homogeneous, interactions with biomolecules will also be affected (*e.g.* binding orientation of surface groups and binding affinity of the probes to their receptor), and thus the interpretation of any biological process mediated by the functionalised probes will be difficult. Therefore, being able to adequately assess the surface functionalisation of a given NP suspension is of the utmost importance.

Nanoparticles can be characterised by myriad methods, including small-angle X-ray scattering (SAXS),^23,24^ dynamic light scattering (DLS),^25,26^ optical waveguides,^27^ microresonators,^28^ and microcavities.^29,30^ Nonetheless, approaches facilitating direct measurements of the surface coverage of nanoparticles are rare compared to the proportion of scientific literature that employs NP functionalisation due to the small size of the inclusions and the high sensitivity required to detect differences in functionalisation.^26^

As surface functionalisation modifies physical properties, the optical properties of the nanoparticle, such as its refractive index (RI), should also change and be characterisable. Refractive index measurements are well established in measuring nanoparticle properties and surface biosensing, being integral to techniques such as surface plasmon resonance (SPR).^31^ The RI of the nanoparticles themselves is utilised extensively in fields such as tailored or tunable optical materials^32–34^ created through doping, as well as optical cloaking.^35,36^ Measurements of nanoparticle RIs have been performed using techniques such as inline holography,^37^ turbidimetry,^38,39^ nanoparticle tracking analysis,^40^ and SPR itself.^31^ Yet, there are downsides to these methods. For example, single particle techniques^37^ are statistically limited whereas absorption-based techniques^38^ suffer from the need for very high volume fractions (∼1%). In addition, many methods, including NTA and holography, have a minimum size below which characterisation is impossible.

Here we propose a simple and robust interferometric technique for the measurement of nanoparticle refractive indices to assess the degree of surface fuctionalisation for carbon dot nanoparticles (CDs) of ∼2 nm diameter, whilst requiring very low sample volumes. This all-optical technique is non-destructive, highly repeatable, and has no explicit minimum particle size. We utilise the principle that a functionalised nanoparticle has a different RI to that of an unfunctionalised one, yielding results that correlate well with estimates from Mie theory. By attributing this RI change to a homogeneous outer layer, a core–shell permittivity model can be used to determine the volume fraction that the coronal shell occupies.

## Refractive index of nanoparticle suspensions

Nanoparticles are typically suspended in a medium, with only the overall refractive index of the mixture 
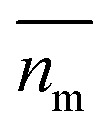
 able to be measured, where the horizontal bar represents a complex variable. For that reason, the relationship between the measured RI of the mixture and the RI of the particles must be elucidated. It was shown by van de Hulst^41,42^ (the full derivation can be found in the ESI, section I†) that the complex refractive index of a mixture containing a mass fraction of particles *c*_p_ with refractive index 
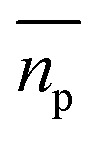
 and density *ρ*_p_ in a medium of RI *n*_l_ (considered to only have a real component here) is represented by1
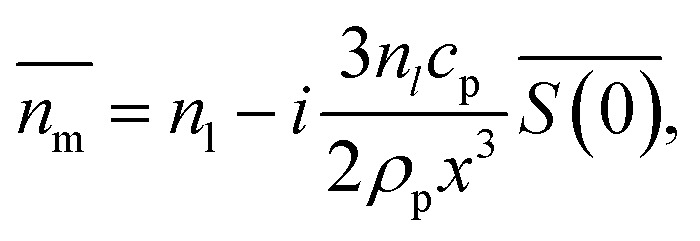
where 
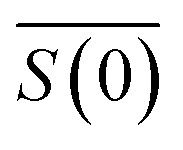
 is the scattering amplitude in the forward direction. Here *x* = 2π*n*_l_*r*_t_/*λ* is the size parameter where *λ* and *r*_t_ denote the vacuum wavelength of the incident light and radius of the particle respectively. In this work, the mass fraction *c*_p_ is defined to be the mass of particles per unit volume and can be related to the particle volume fraction *χ*_p_ (the fraction of a given volume occupied by particles) by *c*_p_ = *χ*_p_*ρ*_p_ = 4/3π*r*_t_^3^*Nρ*_p_, where *N* is the number density of nanoparticles.^43,44^ It is important to note that the density of the nanoparticles is dependent on particle composition, which will be addressed in a later section. The forward scattering amplitude 
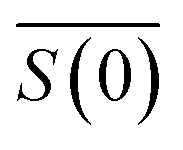
 is a complex function derived from Mie theory and is directly related to the scattering cross-section of a particle which can be numerically calculated for any values of *x*, 
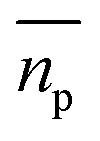
, and *n*_l_. In the case of Rayleigh particles that are small compared to the wavelength of light however, an exact solution for 
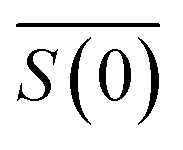
 is available:^45,46^2
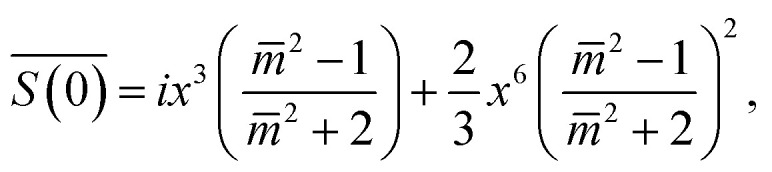
where 
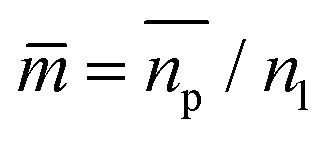
 is the relative refractive index of the particle to the surrounding medium. As for Rayleigh particles *x* ≪ 1, the second term of eqn (2) is negligible compared to the first and can be ignored. By substituting eqn (2) into eqn (1), and dropping the bar notation by assuming the particles have only a real refractive index component, the refractive index of a suspension of Rayleigh particles is found to be3
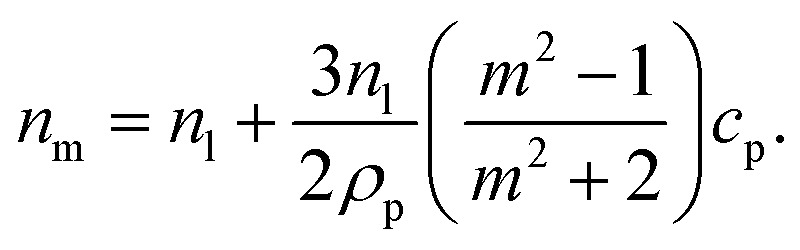


It can be seen from eqn (3) that the refractive index of the suspension is uninfluenced by the size of the particles other than in determining the mass fraction of particles (which can be circumvented by weighing). In addition, eqn (3) is independent of the wavelength of the light, other than small changes in the RI of the materials considered with wavelength. Therefore, to ascertain the RI of the particle, the refractive index increment per unit mass fraction (d*n*_m_/d*c*_p_, often referred to as the refractive index increment)4
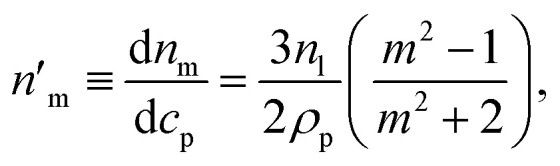
can be measured, which is equivalent to the slope of a graph of suspension refractive index against particle mass fraction.

Eqn (4) shows that the refractive index of a homogeneous Rayleigh particle can be extracted provided the refractive index increment and the refractive index of the medium are measured. In this case, solving eqn (4) for *n*_p_ gives5
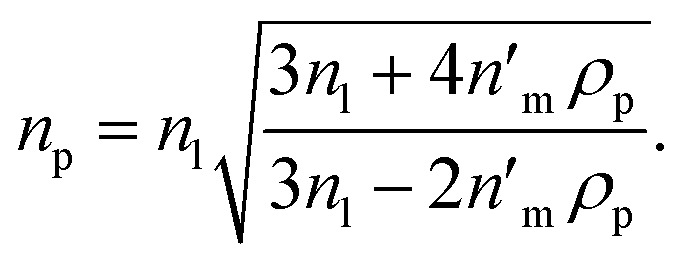


High mass fraction samples are typically not feasible to create, meaning that a very sensitive system to measure the refractive index change is needed. Here backscattering interferometry (BSI) is used to detect refractive index changes on the order of ∼10^−6^ refractive index units (RIU) and determine the particle RI by varying the mass fraction. It is worth noting that “backscattering interferometry” is a misnomer as no backscattering is detected in this configuration; only forward scattering contributes to the refractive index change. However, backscattering interferometry is the accepted term in the literature, so this convention is echoed here.

## Materials and methods

### Backscattering interferometry

**Fig. 1:**
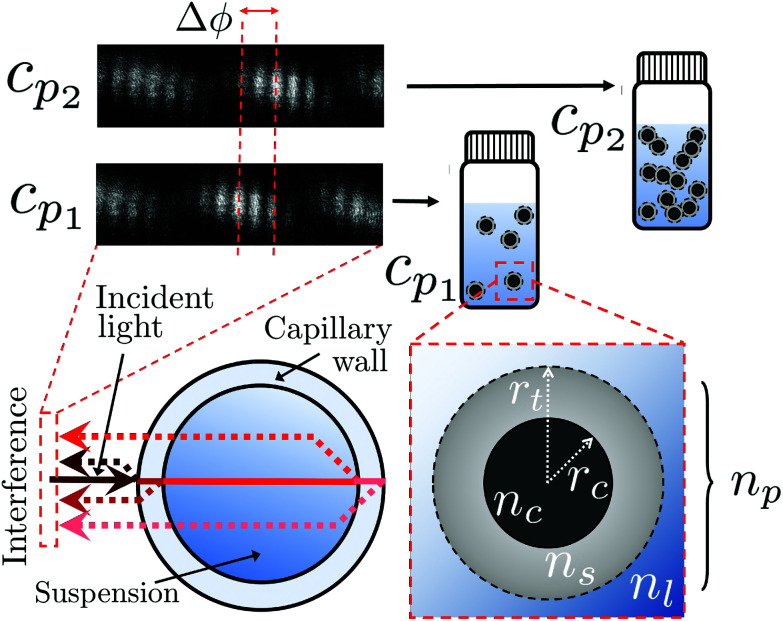
A pictorial representation of the technique. Incident light on a capillary creates a fringe pattern that shifts by Δ*ϕ* depending on the nanoparticle morphology and volume fraction. The nanoparticles in the suspension are modelled as core of radius *r*_c_ and refractive index *n*_c_, surrounded by a shell of outer radius *r*_t_ and refractive index *n*_s_. The full core–shell particle can be considered a single, homogeneous particle of effective refractive index *n*_p_.

In BSI, a fringe pattern is generated by interference of partial reflections at the optical boundaries in a capillary tube, as shown in Fig. 1 and described in greater detail previously.^47^Fig. 1 also shows how the periodic intensity pattern will shift laterally depending on the refractive index of the mixture contained within it (for example *c*_p1_ to *c*_p2_) due to the relative changes in optical path length between the reflected paths. The corresponding phase shift of the fringe pattern Δ*ϕ* is given by^48^6
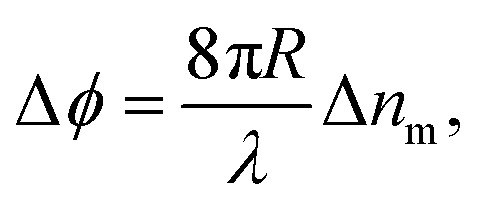
where *R* is the radius of the capillary used and Δ*n*_m_ is the overall change of the refractive index of the suspension (compared to some reference of known refractive index, typically *n*_l_ such that Δ*n*_m_ = *n*_m_(*c*_p_,*n*_p_) − *n*_l_). From eqn (6), it is therefore possible to determine the change in RI of a suspension from a known change in mass fraction. Using eqn (5), both *n*_p_, and subsequently, the degree of functionalisation of the particles can be determined when comparedagainst unfunctionalised particles.

**Fig. 2:**
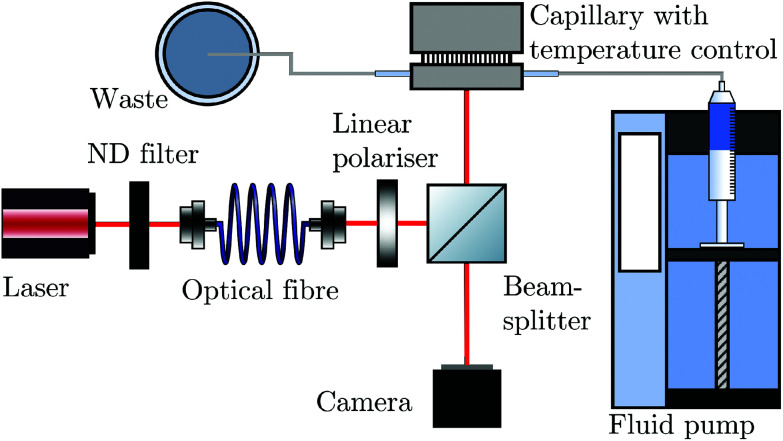
A diagram reproduced from Mulkerns *et al.*^47^ showing the backscattering interferometer used here. A coherent laser beam is incident on a thermally stabilised capillary tube containing the nanoparticle solution, creating an interference pattern due to the reflections at the multiple interfaces of the capillary. This pattern of fringes is then imaged by the camera and transferred to a computer for analysis.

The BSI apparatus used in this article is shown in Fig. 2 and is based on the design used by Bornhop *et al.*^49^ As shown in Fig. 2, a helium–neon laser (JDS Uniphase, 7 mW) is passed through aneutral density filter (ND filter, ThorLabs, FW1AND) and single-mode optical fibre (ThorLabs, S405-XP) to regulate power and beam quality. The polarisation state of the laser is then enforced by a linear polariser (ThorLabs, LPVISE100-A) and the light is directed towards a capillary (Drummond, ID 0.59 mm) by a 50 : 50 beamsplitter (ThorLabs, BSW-10). The capillary creates a common path interferometer where the light interferes with itself due to the reflections and transmissions at the multiple refractive index boundaries of the capillary. The capillary is mounted in a custom-designed stage, held at a constant temperature by a Peltier thermoelectric heater/cooler (Laird annular SH-10 controlled by Wavelength Electronics WTC3243HB) to better than 0.001 °C. The backscattered (*i.e.* the opposite direction to the incident laser light) interference pattern is imaged by a CMOS camera (Ximea MQ013CG-E2) and streamed to a computer (not shown).

### Nanoparticle solutions

Nanoparticle solutions were created from a stock (Phosphorex PMMA Nanospheres, *r*_t_ = 29.7 ± 8.4 nm) by aliquoting with MilliQ pure water, and then vortexed and sonicated for one minute each before being introduced to the capillary slowly by a syringe pump (Cole-Parmer Masterflex) to avoid mechanical instabilities and turbulence. Acid functionalised carbon dots (CDs) were synthesized from citric acid and ethylene diamine following a modified procedure as set out by Mondal *et al.*^50^ Functionalisation with 1-aminoethyl lactose (Lac) or amine-bearing trivalent lactoside (Lac3) *via* EDC-mediated coupling yielded CD-Lac and CD-Lac3 (see ESI, section III† and Materials and methods for full experimental details). Each sample was analysed for a period of 20 seconds after waiting approximately 200 seconds from the point of injection to ensure the temperature of the mixture stabilised. The fast Fourier transform of the fringe pattern is taken and the phase of the lowest frequency peak is tracked over time, after spatial averaging is performed.^51^ This phase shift is tracked as a function of the particle concentration to extract the refractive index change as a function of mass fraction (see eqn (4)), which can be used to ascertain the particle refractive index and functionalisation layer thickness.

## Determining nanoparticle refractive indices

**Fig. 3:**
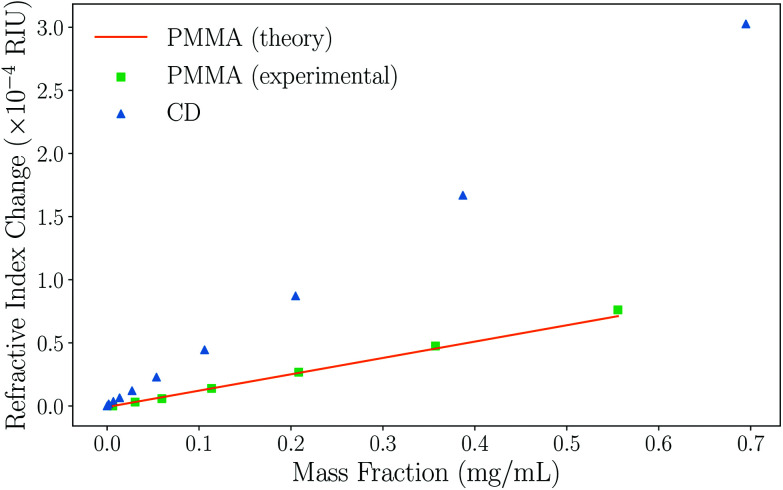
A graph showing how the refractive index of the overall particle suspension varies with mass fraction for Rayleigh PMMA nanoparticles and unfunctionalised carbon dots in water using BSI. The experimental results obtained for PMMA (green squares) and the Mie theory data (orange circles) match well, indicating the ability to measure the refractive index of nanoparticles. The carbon dot data (CD, blue triangles) can be fitted and eqn (5) used to determine their refractive index. Note that error bars are present, but too small to see on this plot; a typical error on a measurement is *σ* ≈ 2 × 10^−6^ RIU.

To demonstrate the suitability of the technique for measuring the refractive indices of Rayleigh particles in suspension, varying dilutions of PMMA nanoparticles in water were measured. Numerical simulations confirm that the PMMA microparticles are comfortably in the Rayleigh regime due to their low RI contrast (see ESI, section II†). Excellent correlation between the experimental data collected and the Mie theory data obtained using the manufacturers stated radius *r*_t_ = 29.7 ± 8.4 nm and the refractive index of PMMA from literature (*n*_p_ = 1.4908)^52^ is observed, as can be seen in Fig. 3. Further validation was performed using polystyrene nanoparticle suspensions, also finding excellent agreement to theoretical predictions. This data can be found in the ESI, section II.†

Based on this high degree of correlation for thoroughly characterised particles with well-defined properties, the refractive index of the unfunctionalised carbon dots (CDs) can be extracted from the data in Fig. 3 using eqn (5). The refractive index of the naked carbon dots is found to be 1.693 ± 0.006, which is used as the refractive index of the core of a functionalised CD in subsequent analysis. Comparison of this data is difficult due to the wide range of physio-chemical structures and associated values given in the literature; for example, the refractive index of thin film amorphous carbon at 632.8 nm has been experimentally determined to be between 1.8849 and 2.5307 according to different sources.^53,54^ Similarly, the density of amorphous carbon has a wide range of accepted values. Here, the density was measured to be 1.9 g cm^−3^ using pycnometry.

**Fig. 4:**
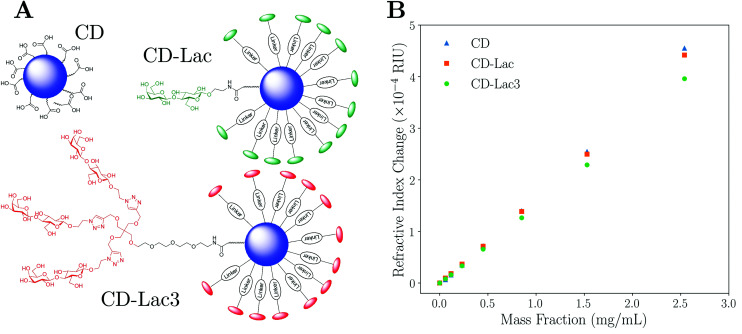
Representations of carbon dot (CD), CD-lactose (CD-Lac), and CD-lactose dendrimer (CD-Lac3) particles are shown in A. B shows a graph of the experimentally measured change in refractive index of the solution as a function of CD mass fraction. Blue triangles refer to data for unfunctionalised CDs, orange squares to CDs functionalised with lactose and green circles are data for CDs functionalised with a lactose-based tri-dendrimer. The difference in the gradients in B shows that the various surface functionalisations can be differentiated easily. The inset of B shows a magnified copy of the data at low mass fraction. Note that error bars are present on all data, but can only be seen in the inset; a typical error on a measurement is *σ* ≈ 2 × 10^−6^ RIU.

A possible explanation for the refractive index of the unfunctionalised carbon dots being lower than one would naively expect could be the presence of small amounts of impurities such as CD precursor materials. These additives would act to artificially increase the refractive index of the surrounding medium, and therefore mean that the RI of the CDs is underestimated. However, when samples were analysed using Atomic Force Microscopy (AFM), little evidence of impurities was found (see Fig. S24 in ESI†) strongly suggesting that the RI determined here is the true value. In addition, as all CD samples were purified similarly, any effect on the data (including functionalised particles) should be approximately systematic and therefore negligible when data are compared. The nanometre size of the nanoparticles could also cause the bulk and nanoscale properties to diverge. However, to the best of our knowledge, any size-mediated effects on the RI or density of carbon nanoparticles have not been documented. Lastly, the presence of unavoidable, very low molecular weight functional groups on the surface of the unfunctionalised CDs (see Fig. 4A and ESI, section III†) would act to reduce the refractive index of the CDs slightly compared to bulk carbon. The experimental value for the RI of naked carbon dots can be subsequently used to determine the thickness of the functionalised layers present on other CDs.

## Nanoparticle functionalisation thickness

Characterisation data for lactose-functionalised CDs and lactose dendrimer-functionalised CDs (CD-Lac and CD-Lac3 respectively) were taken as set out in the Materials and methods section. Fig. 4A shows difference in surface functionalisation for the three CD samples, with Fig. 4B showing how the refractive index of the carbon dot suspension varies with mass fraction for naked CDs, CD-Lac and CD-Lac3. There is a clear difference in the refractive index increment (d*n*_m_/d*c*_p_) of the graphs of these three nanoparticles, unequivocally demonstrating that differences in surface functionalisation can be detected by this technique. From the inset in Fig. 4B, an estimate of the minimum mass fraction needed to discern the difference in surface functionalisation can be obtained from the point at which the refractive index difference becomes commensurate with the uncertainty on a single measurement; approximately *c*_p_ ≈ 0.2 mg mL^−1^. As the illuminated volume of the capillary is only ∼2 μL, the amount of sample required for this technique is a fraction of that used in other techniques such as SAXS^23,24^ or turbidimetry.^38,39^ For example, turbidimetry requires volume fractions on the order of ∼1%, whereas here the volume fractions required to distinguish the CDs are ∼0.01%. This limit of detection will scale both with the difference in volume of the functionalisation layer and the particle radius; it is much easier to determine a difference in gradient for a particle with a large corona to core volume ratio. Additionally, the limit of detection will depend upon the apparatus used, the capillary radius, and the wavelength of light used.^47^ Therefore, the limit of detection will only be exact for the combination of parameters stated in this text.

To quantitatively assess the degree of functionalisation, the effective refractive index of each functionalised particle type can be determined by fitting a straight line and utilising eqn (4). For the data in Fig. 4B, the average refractive indices for CD-Lac and CD-Lac3 are determined to be 1.662 ± 0.005, and 1.591 ± 0.003 respectively. The errors on these values are dominated by the error on the linear fit when determining d*n*_m_/d*c*_p_, explaining why they are not the same. The reduction in the refractive index of the particle is due to the functionalisation layer reducing both the average refractive index and density of a single particle. To quantify this effect, a relationship between the thickness of the molecular corona and the refractive index of the particle must be established.

To a first approximation, the corona of molecules that surround a particle can be modelled as a spherical shell of outer radius *r*_t_ and refractive index *n*_s_^55,56^ surrounding a core with inner radius *r*_c_ and refractive index *n*_c_, as is shown in Fig. 1. Using this assumption, a core–shell model particle can be “internally homogenised”,^35,41,57^ allowing it to be considered a uniform particle of radially-independent effective refractive index *n*_p_ such that7
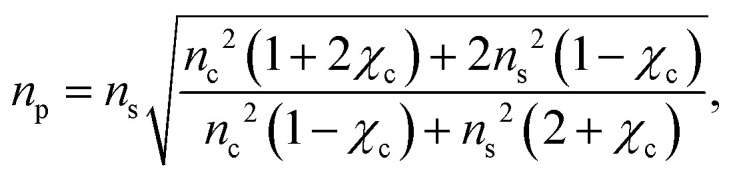
where *χ*_c_ = (*r*_c_/*r*_t_)^3^ is the volume fraction of the particle associated with the core and it is assumed that *n*^2^ = *ε*, where *ε* is the relative permittivity (*i.e.* the materials are non-magnetic). By rearranging eqn (7), the volume fraction parameter can be extracted and is found to be8
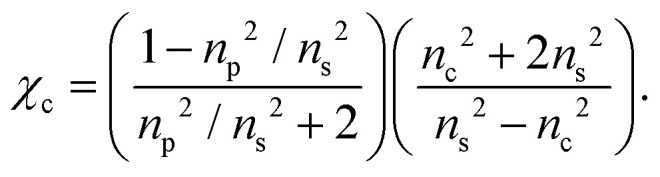


As shown in eqn (4), extracting the refractive index of the particle is dependent on knowing the true density of the particle. In the special case of unfunctionalised nanoparticles, the particle density is simply equal to the core density *ρ*_c_. However, if a shell is present, then the particle density will be given by a volume-weighted sum of the shell and core densities *ρ*_s_ and *ρ*_c_ respectively^58^

9 *ρ*_p_ = *χ*_c_*ρ*_c_ + (1 − *χ*_c_)*ρ*_s_ = (*ρ*_c_ − *ρ*_s_)*χ*_c_ + *ρ*_s_.

In this work, the density of the core and shell are assumed to be *ρ*_c_ = 1.9 g cm^−3^ and *ρ*_s_ = 1.52 g cm^−3^. If a shell is present, eqn (4), (8), and (9) are coupled and can be combined to yield10
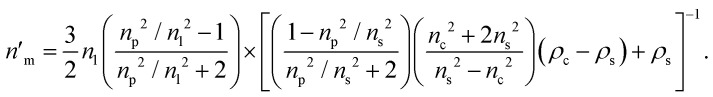


By rearranging eqn (10) using a symbolic solver (Sympy version 1.1.1) to determine *n*_p_, it is possible to calculate the thickness of the corona using eqn (8). In doing so, it is assumed that the refractive index of the shell is the same as that of pure, ordered lactose for both CD-Lac and CD-Lac3 (*n*_s_ = 1.5663),^59^ and also that the composition of the core of the carbon dots is consistent for each sample such that the refractive index of naked CDs can be used as the core refractive index for the functionalised samples. The chemical forms of the surface moieties for CD-Lac and CD-Lac3 are not identical (or simply a copy) due to differences in linkage (see Fig. 4A), but the differences are minor and the approximation of a similar refractive index is therefore reasonable. Medium angle X-ray scattering (MAXS, see ESI, section V†) and atomic force microscopy (AFM, see ESI, section IV†) were employed to independently determine the radii of the functionalised and unfunctionalised carbon dots. Using MAXS, the radii for naked CDs, CD-Lac and CD-Lac3 were determined to be 9.8 ± 0.2 Å, 11.5 ± 0.1 Å and 16.0 ± 0.1 Å (shell thicknesses of 1.7 ± 0.2 Å and 6.1 ± 0.2 Å respectively). AFM data were taken for unfunctionalised CDs and CD-Lac3, finding radii of 9.6 ± 2.4 Å and 17.0 ± 1.2 Å (shell thickness of 7.4 ± 2.7 Å), which compares very well with the values found by MAXS. Using eqn (8) and (10) combined with the knowledge of the average core radius from AFM and MAXS, the size of the functionalised layer experimentally determined here is found to be 0.9 ± 0.2 Å and 7.0 ± 0.6 Å respectively for CD-Lac and CD-Lac3. The experimental values found here are similar to those determined by both MAXS and AFM. It should be noted, however, that the errors given here are an underestimation as the systematic errors common to all measurements have been omitted to aid internal comparison. This is in part due to the inability to verify some presuppositions such as the RI or density of lactose and the lactose dendrimer around the particles being the same and of the value assumed here.

One potential source of the discrepancy between the shell thickness found by BSI, AFM, and MAXS is the fact that this shell thickness measured is an ensemble average, meaning that it may be the case that the functionalisation layers are only partially filled, leading to a measured average thickness lower than that expected. If this were the case, it would mean that this technique allows measurement of sub-monolayer coverage, a characteristic that is typically impossible to measure with many other techniques.^25^ Higher surface curvature (*i.e.* smaller nanoparticles) typically favours greater surface coverage due to the minimisation of steric effects.^25^ However, even a fully functionalised nanoparticle will not have moieties that cover the full surface area due to steric hindrance, leading to shell thickness underestimation as it is assumed to be homogeneous.

As the thickness is highly dependent on the radius of the particle as shown in eqn (7), an error in this parameter would highly influence the final thickness. This is especially true as the radius of nanometre-sized particles is notoriously difficult to determine with accuracy, as shown by both the MAXS and AFM data. Furthermore, there is some degree of polydispersity in these carbon dots due to the difficulty in controlling this factor during synthesis. Based on this, the ensemble measurement will be dominated by small particles (those with high shell–volume ratios) if a constant functionalisation thickness is assumed. Differences in chemical bonding or hydration between the samples may also lead to greater uncertainty in the data, but there currently does not exist a satisfactory technique to clarify this.^56^ It should be noted that any size uncertainty, polydispersity, or other effects such as aggregation will not affect the final refractive index measurements (see eqn (4) and (5)), in the limit that these effects are not large enough to push the particles outside of the Rayleigh regime. Given that AFM data showed negligible aggregation, and multiple techniques recorded similar ensemble radii, any size-mediated discrepancies can be ignored in this case.

The data provided here demonstrates the ability of this interferometric technique to distinguish between different nanoparticle surface moieties in their natural media, whilst requiring a very low sample mass. While investigated here for dielectric Rayleigh particles, there is no reason that this technique could not be extended to encompass particles in the Mie regime, to metallic core nanomaterials such as gold nanoparticles, and to particles where the payload is instead carried in the core (*e.g.* vesicles).

## Conclusions

An all-optical technique for determining the refractive index of nanoparticles in suspension has been experimentally verified. Subsequently, it was shown that, using a modified backscattering interferometer, the coronas of lactose and lactose dendrimers on nanometre-sized carbon dots can be distinguished through their differing respective contribution to the effective refractive index of the particle. The process intrinsically determines an ensemble average, not requiring a statistical picture to be builtup over time. It also does not necessitate high particle volume fractions, is non-destructive, and requires no labelling. We believe that this technique would be an excellent characterisation tool for both dielectric and metallic nanoparticles in optically transparent media, especially as the technique can be deployed in-line. Given the enthusiastic adoption in scientific literature of drug-loaded nanoparticles as theranostic devices and lack of quantitative methods for determining the degree of functionalisation, the technique shown here provides clear advantages for biological applications where rapid assessment of functionalisation is paramount.

## Experimental

### Backscattering interferometry measurements

Backscattering interferometry measurements were taken using an experimental apparatus set out in Fig. 2. PMMA Nanoparticle mixtures were created by dilutions of stock to the required concentrations in glass vials, then mixed by approximately 30 s of sonication and vortexing to ensure homogeneity. CD samples were created by weighing out the correct amount of lyophilised powder in a glass vial, then re-hydrating this stock to the required mass concentration with Milli-Q water. Dilutions of this stock with the same solvent were created and mixed by sonication and vortexing. All samples were left to equilibrate in the laboratory for at least 1 hour before use in the BSI apparatus to ensure a consistent temperature. Samples were introduced to the capillary by a syringe pump at low speed (0.5 mL min^−1^) at an excess (∼2 mL) to ensure any longitudinal mixing did not influence the mixture being probed in the focal volume. Each mixture was allowed to equilibrate with the stage for 200 seconds before a 20 seconds measurement of the phase was recorded at 5 measurements per second with a 100 pixel averaging as set out previously.^51^ The mean of each phase value was taken and converted to refractive index using eqn (6). The capillary was flushed between experiments to remove any residue.

### Synthesis of carbon dots

To manufacture the acid-coated CDs, a protocol similar to that set out previously^12^ was used. In brief, citric acid (1 g, 5.2 mmol) was dissolved in distilled H_2_O (10 mL) in a 250 mL conical flask. Ethylenediamine (EDA, 384 μL, 5.72 mmol) was then added to the solution and stirred for 30 min to ensure homogeneity. The conical flask was then placed in a domestic 300 W microwave and the solution was irradiated for 10 min. A viscous amber residue was obtained which was washed with a 1 : 1 solution of MeOH : acetone four times. The precipitate was then phase-separated by centrifugation and re-dissolved in 15 mL of distilled H_2_O. The CD solution was purified *via* centrifuge-filtration using Vivaspin concentrators (MWCO 10 kDa, 8500 rpm, 30 min). The supernatant was concentrated *in vacuo* (or lyophilised) to yield powdered carbon dots. Functionalisation of acid coated CDs with an excess of lactose disaccharide (Lac, compound 7 in ESI†) or tri-armed lactose disaccharide dendrimer (Lac3, compound 8 in ESI†), which bear linkers terminated with an amine group for amide conjugation, was carried out using hexafluorophosphate azabenzotriazole tetramethyl uronium (HATU) in *N*,*N*-dimethylformanide (DMF) as the solvent (see ESI, section III†). The final structures for CD, CD-Lac, and CD-Lac3 nanoparticles are shown in Fig. 4A. NMR confirmed the surface functionalisation of the CDs to yield CD-Lac and CD-Lac3 (see ESI, section III† for full synthesis and characterisation details).

## Conflicts of interest

There are no conflicts to declare.

## Supplementary Material

NR-014-D2NR00120A-s001
