# Supplementary material for: Measuring the refractive index and sub-nanometre surface functionalisation of nanoparticles in suspension
Source: Nanoscale. 2022 May 5;14(22):8145–52. doi: 10.1039/d2nr00120a (PMC9178438; doi:10.1039/d2nr00120a)
Supplement: NR-014-D2NR00120A-s001 [file NR-014-D2NR00120A-s001.pdf]

Electronic Supplementary Material (ESI) for Nanoscale.
This journal is © The Royal Society of Chemistry 2022

Supporting Information

Measuring the Refractive Index and Sub-Nanometre Surface Functionalization of Nanoparticles in Suspension

Niall M. C. Mulkerns [a,b], William H. Hoffmann [a,b,c], Javier Ramos-Soriano[c], Noelia de la Cruz[c], Teodoro Garcia-Millan[a,c], , Robert L. Harniman[c], Ian D. Lindsay [a,b], Annela M. Seddon [a,b], M. Carmen Galan[c] and Henkjan Gersen[a,b]*

[a]*H. H. Wills Physics Laboratory, University of Bristol, Bristol, BS8 1TL, United Kingdom*

[b]*Bristol Centre for Functional Nanomaterials, University of Bristol, Bristol, BS8 1TL, United Kingdom*

[c]*School of Chemistry, University of Bristol, Bristol, BS8 1TS, United Kingdom*

[*]*Corresponding author: h.gersen@bristol.ac.uk*

I – Derivation of Equation 1

As written by van de Hulst [1], the complex refractive index ratio of a suspension $\bar{m} = \bar{n}_p/n_l$ that contains a number density of scatterers N is given by

$$\bar{m} = 1 - \frac{2\pi i S(0) N}{k^3},$$

where $k = 2\pi n_l/\lambda$, n_l as the medium refractive index (which may be complex, but considered not to be here), $\bar{n}_p$ is the complex particle refractive index, and λ is the wavelength of

illumination. As the size parameter is given by $x = kr_t$, where r_t is the radius of the particle, meaning the equation for $\bar{m}$ can be rewritten

$$\bar{m} = 1 - \frac{2\pi i S(0) r_t^3}{x^3}.$$

As the mass fraction (mass of particles per unit volume) can be written

$$c_p = \frac{4}{3}\pi r_t^3 N \rho_p,$$

where ρ_p is the density of the particles. Therefore, we arrive at Equation 1 in the main text by substitution for N and explicitly stating in terms of n_p:

$$\bar{n}_p = n_l - i\frac{3n_l c_p}{2\rho_p x^3}\bar{S}(0)$$

.

[1] H. C. van de Hulst, *Light scattering by small particles*, **1981**, Courier Corporation.

II - Scattering Simulations

Light scattering simulations for poly(methyl methacrylate) nanoparticles were performed by numerically solving equation 4 of the main text without the assumption that the particles are in the Rayleigh regime. Typical values for PMMA particles dispersed in water were used; $n_l = 1.3315$, $n_p = 1.4908$, $\rho_p = 1.19$ g/cm³. The graph of refractive index increment $\frac{dn_m}{dc_p}$ as a function of particle radius is displayed in Fig. S1. The dashed red line indicates the size of the PMMA nanoparticles used in this manuscript, proving that they lay within the Rayleigh regime where the refractive index increment of the mixture is size-independent. All simulations were written in Python using the package PyMieScatt for evaluation of the complex forward scattering coefficient $\bar{S}(0)$.

Additional data were taken with polystyrene nanoparticles (Phosphorex, 29 nm diameter) to test the validity of equation 4 in the main text. These data are displayed in Figure S2; the refractive index calculated from the line of best fit is 1.603, which is in good agreement with the known value of approximately 1.59 [1]. Despite polystyrene being closer in refractive index to the carbon dot systems, polystyrene can exhibit intrinsic optical anisotropy, with a slightly higher refractive index core compared to the outer layers due to manufacturing methods [2,3]. This makes polystyrene a potentially less optimal control compared to PMMA.

[1] X. Ma, J. Q. Lu, R. S. Brock, K. M. Jacobs, P. Yang, X. H. Hu, *Phys. Med. Biol.*, **2003**, 48, 4165–4172.

[2] C. Minelli, R. Garcia-Diez, A. E. Sikora, C. Gollwitzer, M. Krumrey, & A.G. Shard, *Surface and Interface Analysis*, **2014**, *46*(10-11), 663-667.

[3] R. Grunder, G. Urban, & M. Ballauff, *Colloid and Polymer Science,* **1993***,* 271.6, 563-572.

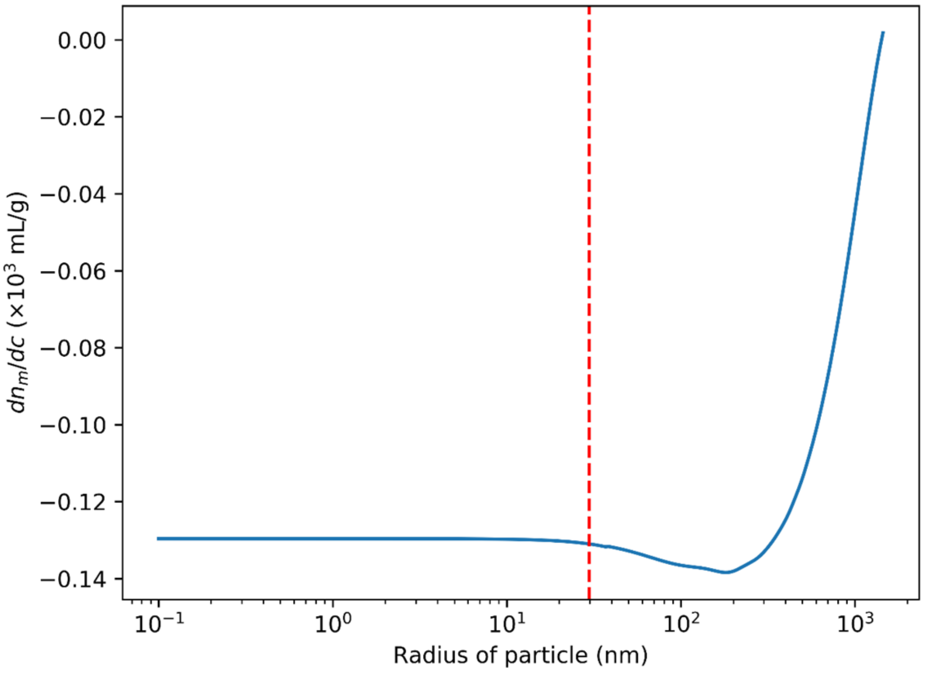

Figure S1: A graph showing the evolution of the refractive index increment as a function of particle radius for PMMA nanoparticles. The dashed line indicates the radius of the particles used in this paper.

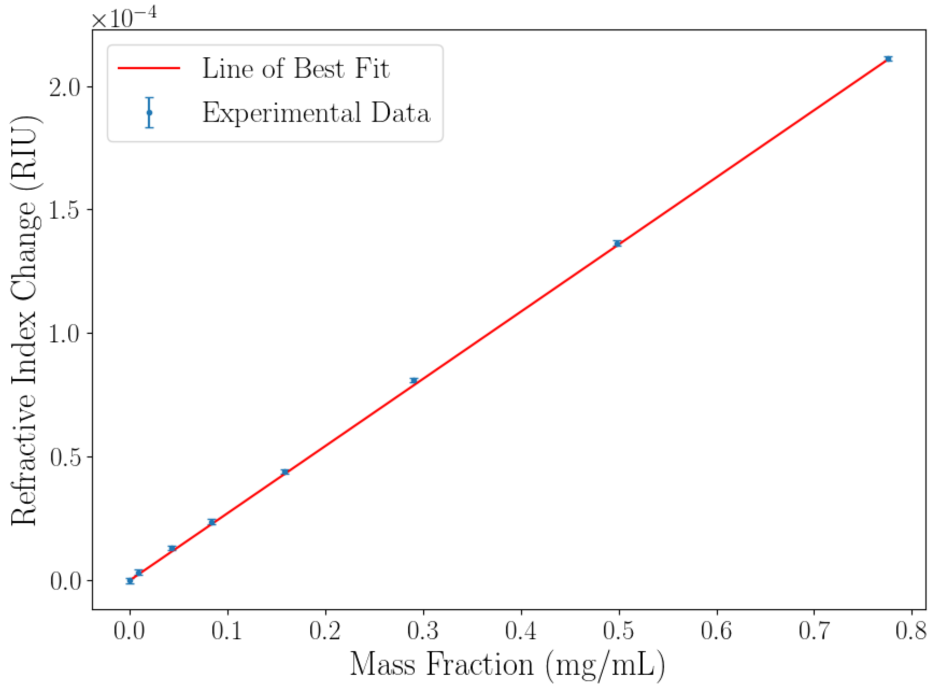

Figure S2: A graph showing the refractive index change measured with varying mass fraction of 15 nm radius polystyrene nanoparticles.

III - General synthesis and characterization of core CDs and functionalised CDs

The list of functionalised carbon dots is shown in Table A. CD-Lac consists of a carbon dot coated with lactose moieties via a two-carbon linker via an amide functionality. Similarly, CD-Lac3 is an identical carbon dot functionalised with a three-pronged dendrimer of lactose molecules, conjugated to the CD via amide conjugation using a PEG linker.

Table S1: A table displaying the functional forms of the three carbon dot samples used.

Name	Structure
CD	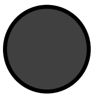
CD-Lac (**7**)	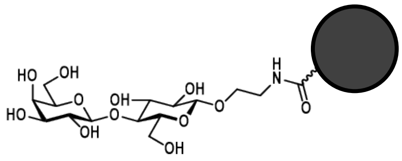
CD-Lac3 (**8**)	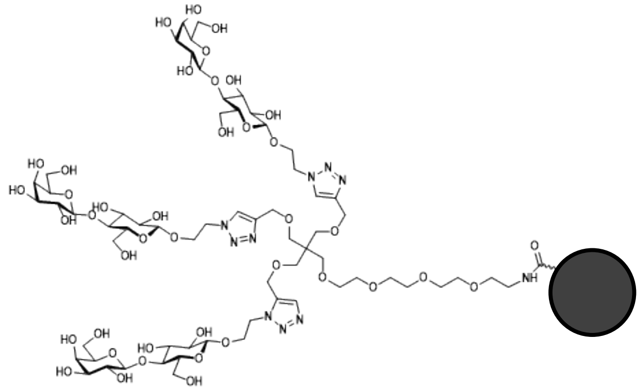

1. General Experimental

Reagents and solvents were purchased as reagent grade from Sigma Aldrich or Fisher and used without further purification. Compounds **1**,[1] **2**,[2] **4**[3] and **5**[4]Error! Bookmark not defined. were prepared according to previously reported procedures. For column chromatography, silica gel 60 (230-400 mesh, 0.040-0.063 mm) was purchased from E. Merck and for gel filtration Sephadex G-25 from GE Healthcare. Thin Layer Chromatography (TLC) was performed on aluminium sheets coated with silica gel 60 F254 purchased from E. Merck, visualization by UV light. NMR spectra were recorded on a Bruker AC 400 with solvent peaks as reference. ^{1}H and ^{13}C NMR spectra were obtained for solutions in $CDCl_3$, CD_3OD and D_2O. All the assignments were confirmed by one- and two-dimensional NMR experiments (DEPT, COSY, HSQC and HMBC). To confirm successful functionalisation of **5** and **6** on the CDs, Diffusion-Ordered NMR Spectroscopy (DOSY), which probes the diffusion coefficient for each of the components of the ^{1}H-NMR spectrum, was acquired. Mass spectra were obtained by the University of Bristol mass spectrometry service using electrospray ionisation (ESI). Microwave irradiation experiments were performed using a Monowave 300 (Anton Pear) apparatus. The temperature in the sealed reaction vessel was monitored by an external surface sensor. Zeta

potential analysis was carried out using Malvern Instruments Nano-Z ZEN 2600 and conducted in distilled H_2O at a concentration of 1 mg·mL^{-1}.

The estimation of the hydrodynamic radii of the CDs (σ^{CD}) was calculated from the DOSY experiments and relative to the residual peak of D_2O, using a simplified relationship of the Stoke-Einstein equation (S1). This simplification allows us to ignore differences of viscosity in the sample.[4]

$$\sigma^{CD} = \frac{D^{w}}{D^{CD}}\sigma^{w} \qquad (S1)$$

Where D^w and D^{CD} are the experimental diffusion coefficients of water (2.3 × 10−9 m2/s) and the CDs and σ^w the radius of water (0.1375 nm).

X-ray photoelectron spectroscopy (XPS) was performed using a Kratos Axis Ultra DLD system, using monochromatic Al *Kα* X- ray source operating at 140 W power (10 mA x 14 kV). Data was collected with pass energies of 160 eV for survey spectra, and 20 eV for the high-resolution scans with step sizes of 1 eV and 0.1 eV, respectively. Samples were pressed on to doubled sided Scotch tape (type 665). The system was operated in the Hybrid mode, using a combination of magnetic immersion and electrostatic lenses and acquired over an area approximately 300 700 μm^2. A magnetically confined charge compensation system was used to minimize charging of the sample surface, and all spectra were taken with a 90° take of angle. A base pressure of ~ 1×10^{-9} Torr was maintained during the collection of the spectra. Data were analysed using CasaXPS (v2.3.23) after subtraction of a Shirley background and using modified Wagner sensitivity factors as supplied by the manufacturer.

2. Synthesis of fluorescent carbon dots (CDs)

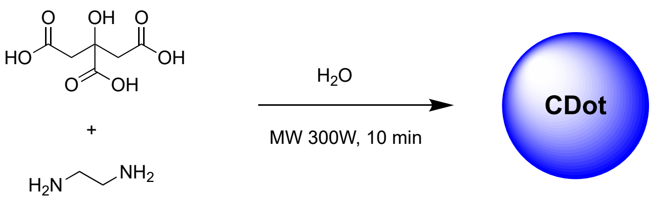

Scheme S1. General synthesis of fluorescent carbon dots.

Procedure modified from Amdursky et al.[5] Citric acid (1.00 g, 5.2 mmol) was dissolved in distilled H_2O (10 mL) in a 250 mL conical flask. Ethylenediamine (EDA, 384 µL, 5.72 mmol) was then added to the solution and stirred for 30 min to ensure homogeneity. The conical flask was then placed in a domestic microwave 300 W (inside a fume cupboard) and the solution was reacted for 10 min. A viscous amber residue was obtained which was washed with a solution MeOH:Acetone 1:1 ($\times 4$). The residue was then phase-separated by centrifugation and re-dissolved in 15 ml of distilled H_2O. The CD solution was dialysed in H_2O using 0.5-01 kDa MWCO Biotech Cellulose Ester membrane. The concentrate CD solution was then concentrated under reduced pressure (or lyophilised) to yield 1.1 g of CD as an amber powder.

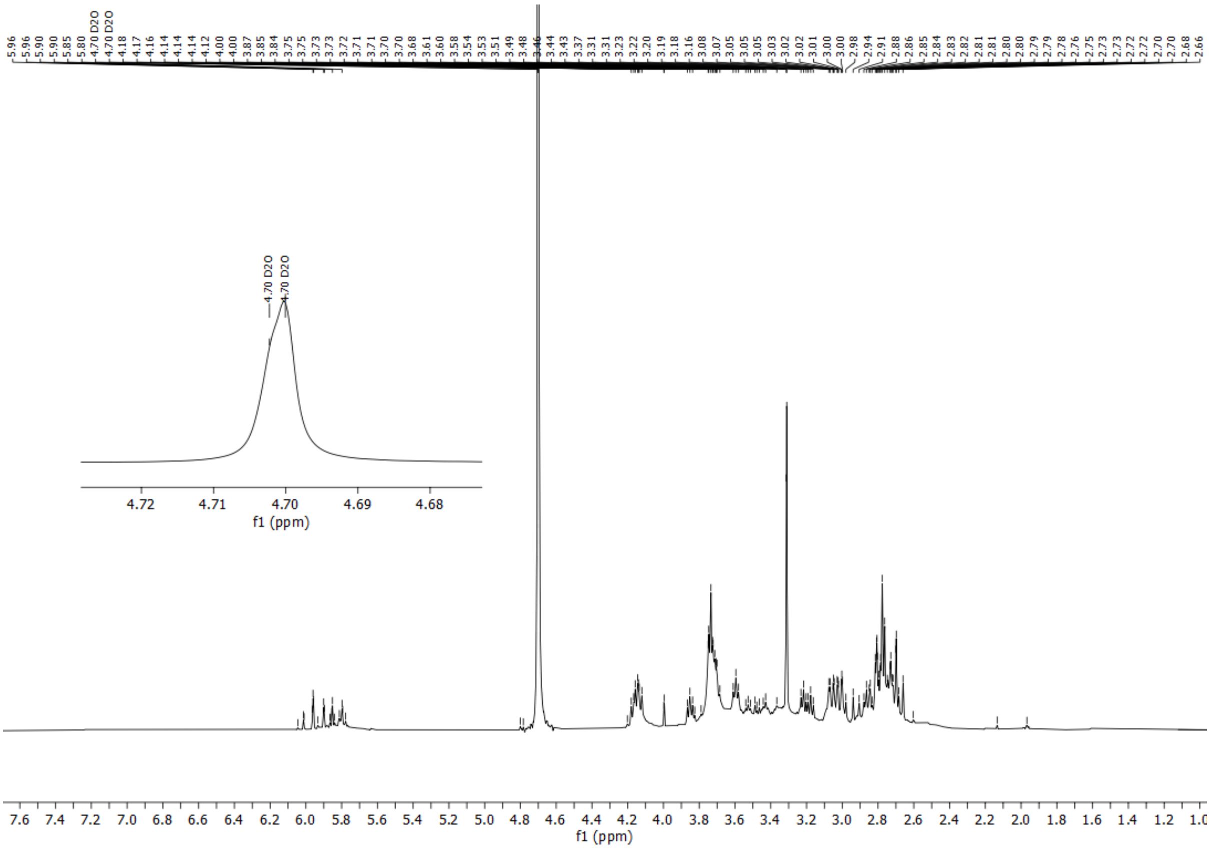

Figure S3: ^{1}H NMR spectrum of CDs (D_2O, 400 MHz).

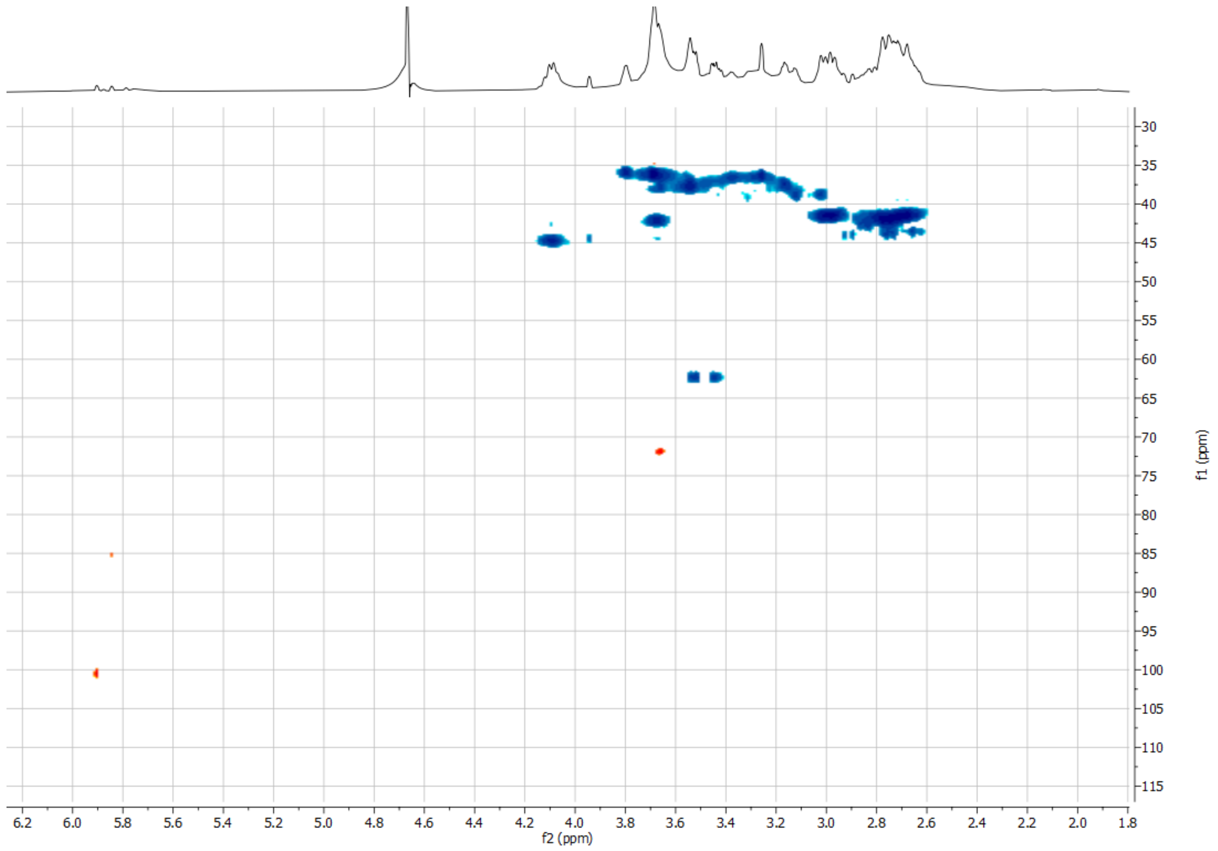

Figure S4: ^1H-^{13}C HSQC NMR (D_2O) spectrum of CD.

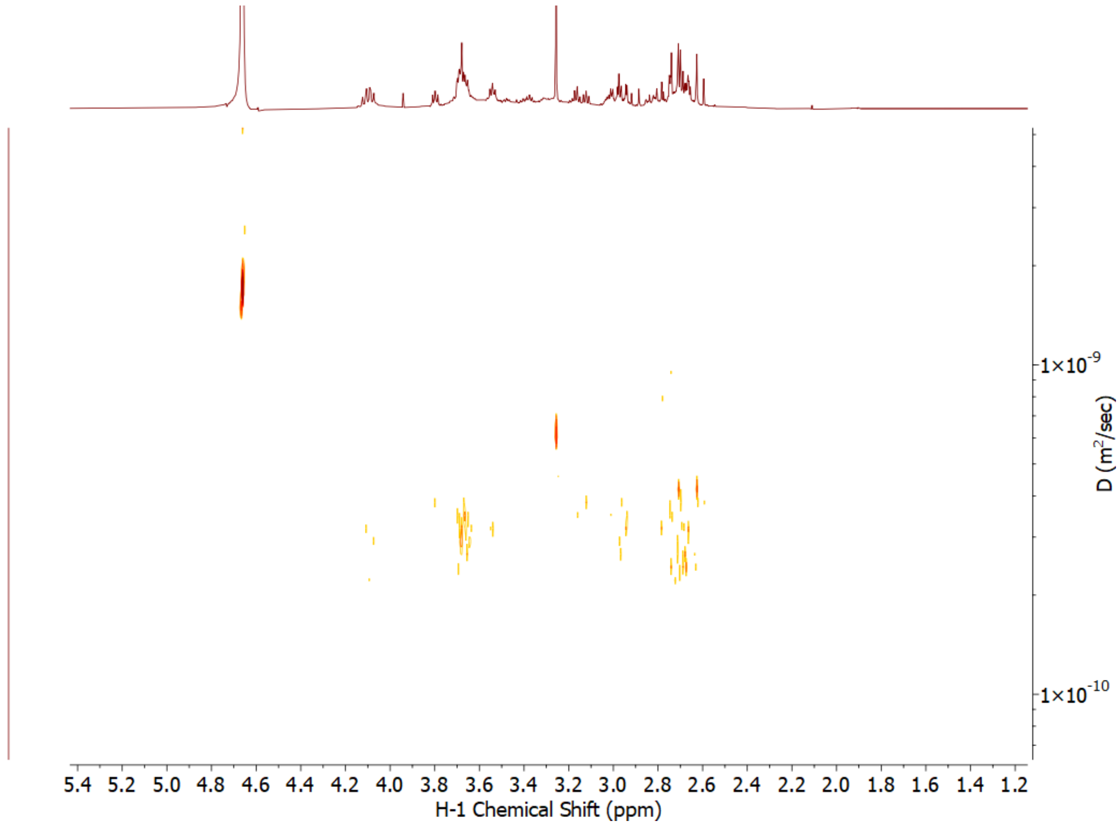

D_w ($m^2 s^{-1}$)	D_{CD} ($m^2 s^{-1}$)	σ_{CD} (nm)
1.71 x 10^{-9}	2.8 x 10^{-10}	0.84

Figure S5: DOSY and ^{1}H NMR spectra of CD.

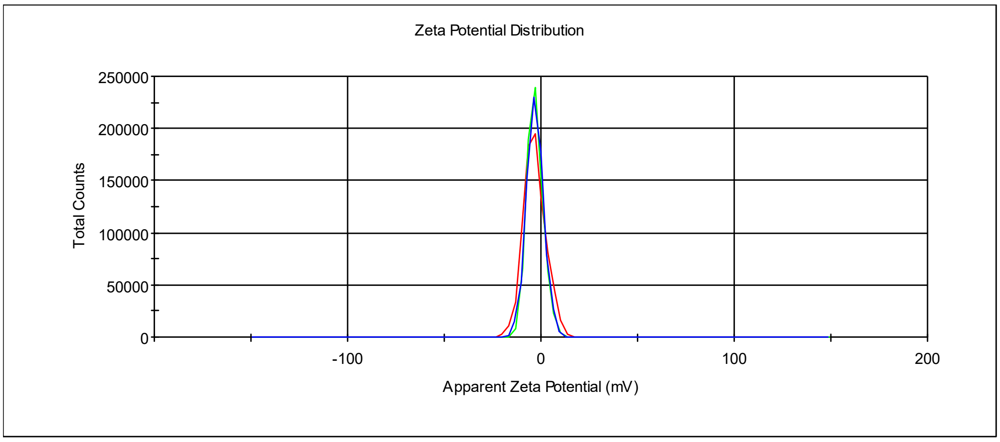

-3.09 ± 0.12 mV

Figure S6: Zeta-potential measurement of CD. Each line represents individual measurements of the same sample, which were used to obtain an average value.

CD	Binding Energy Max / eV	Elemental composition / %	Deconvolution Functionality
C 1s	281.21	12.65	C-C/C=C
C 1s	281.97	15.1	C-COO
C 1s	282.99	17.06	N-C/O-C
C 1s	284.79	18.08	O=C/O-C=O
N 1s	396.74	11.88	C-NH_2
N 1s	398.15	1.61	Imide/graphite N
O 1s	527.66	7.77	C=O/O-C=O
O 1s	528.6	13.66	C-OH/C-O-C
O 1s	529.9	2.2	O=C-O

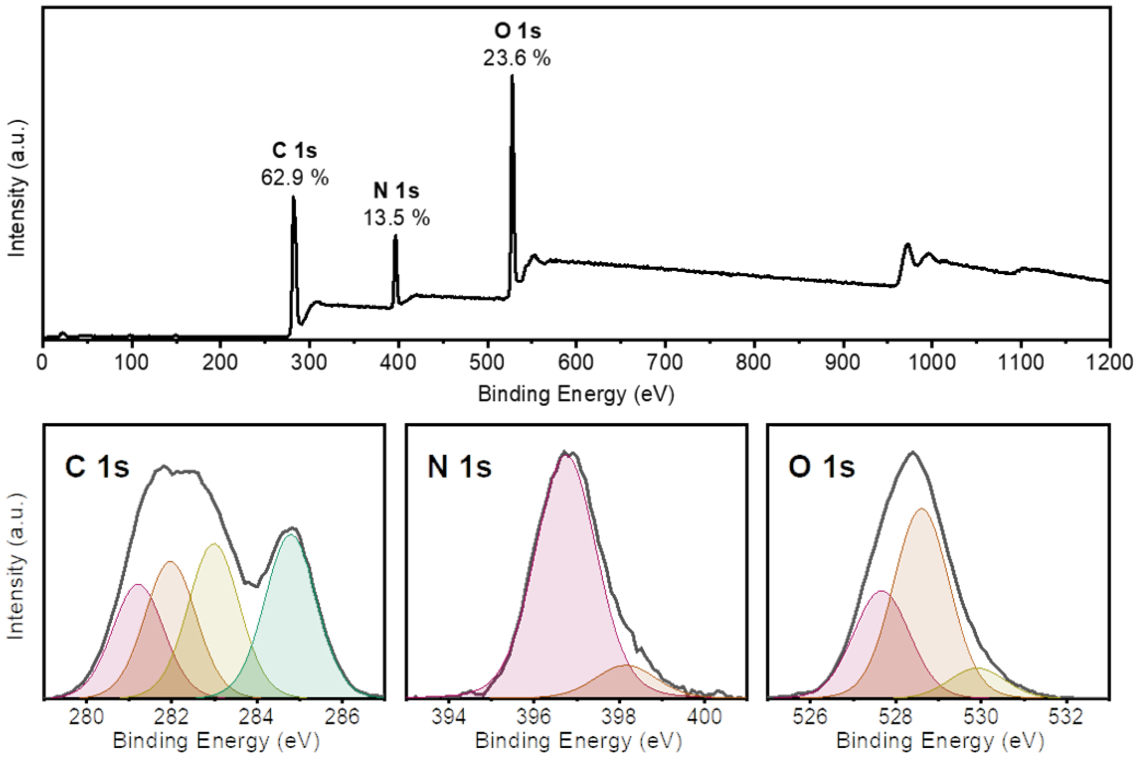

Figure S7: Survey XPS spectrum and narrow scans with deconvolution of the C 1s, N 1s, and O 1s regions of CD.

Table S2: Narrow XPS peak positions, elemental compositions and functionality. Signals referenced to the hydrocarbon peak at 281.21 eV.

3. Synthesis of lactose dendron 5.

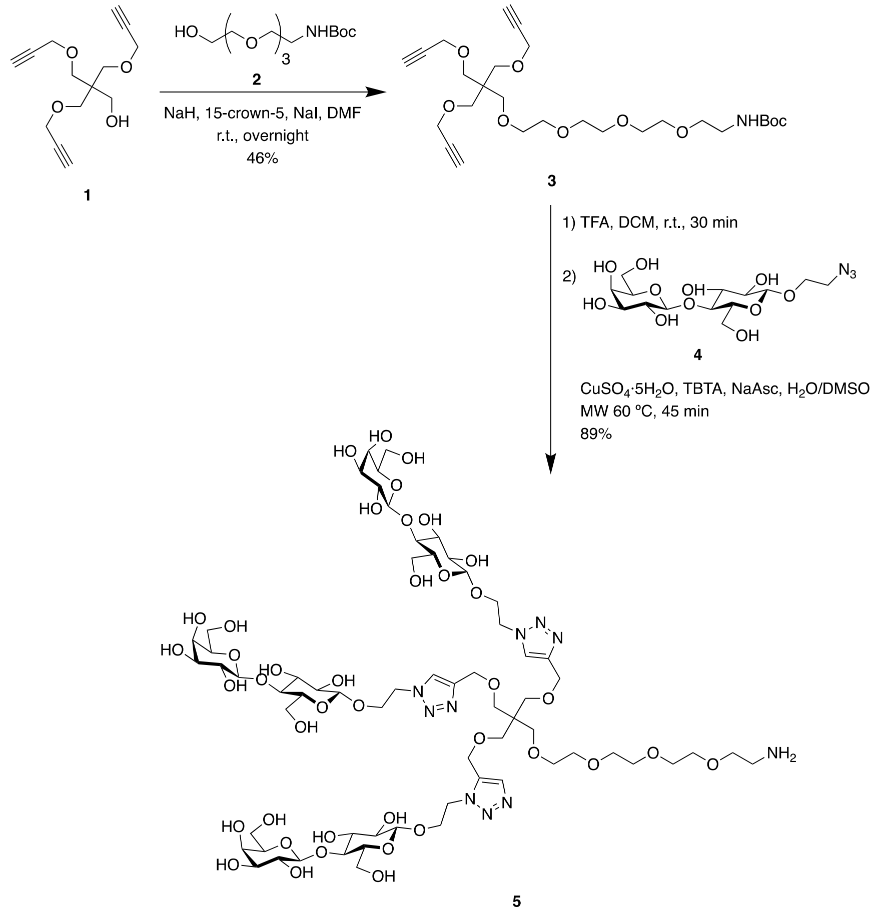

Scheme S2: General synthesis of lactose dendron **5**

Compound 3.

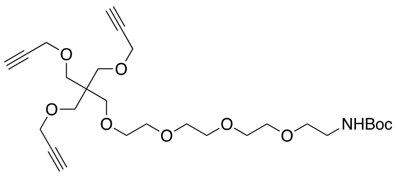

Scheme S3: Compound **3**

Compound **1** (373 mg, 1.49 mmol) was dissolved in dry DMF (4 mL) and NaH (60%, 120 mg, 2.98 mmol) as well as 15-crown-5 (3.4 mg, 0.015 mmol, 1%) were added under nitrogen and stirred at room temperature for 30 min. Subsequently a solution of compound **2** (1 g, 2.24 mmol) and NaI (112 mg, 0.75 mmol) in dry DMF (5 mL) was added. The reaction was stirred overnight at room temperature. After careful addition of MeOH (5 mL) the solvent was removed *in vacuo*. The residue was dissolved in DCM and washed with water. The organic

layer was dried over anhydrous MgSO4, filtered and concentrated. The residue was purified by flash silica chromatography (EtOAc/hexane 2:3 -> 1:1), afforded compound **3** (360 mg, 46%) as a colourless oil. ^{1}H NMR (400 MHz, $CDCl_3$) δ 5.03 (s, 1H), 4.11 (d, J = 2.4 Hz, 6H), 3.69 – 3.54 (m, 14H), 3.52 (s, 6H), 3.45 (s, 2H), 3.31 (m, 2H), 2.40 (t, J = 2.4 Hz, 3H), 1.44 (s, 9H); ^{13}C NMR (101 MHz, $CDCl_3$) δ 80.2, 74.2, 71.1, 70.8, 70.7, 70.6, 70.4, 70.3, 70.3, 69.9, 69.1, 58.7, 45.0, 28.5; ESI-HRMS for $C_{27}H_{44}NO_9$ $[M + H]^+$ calcd: 526.3016, found: 526.3011.

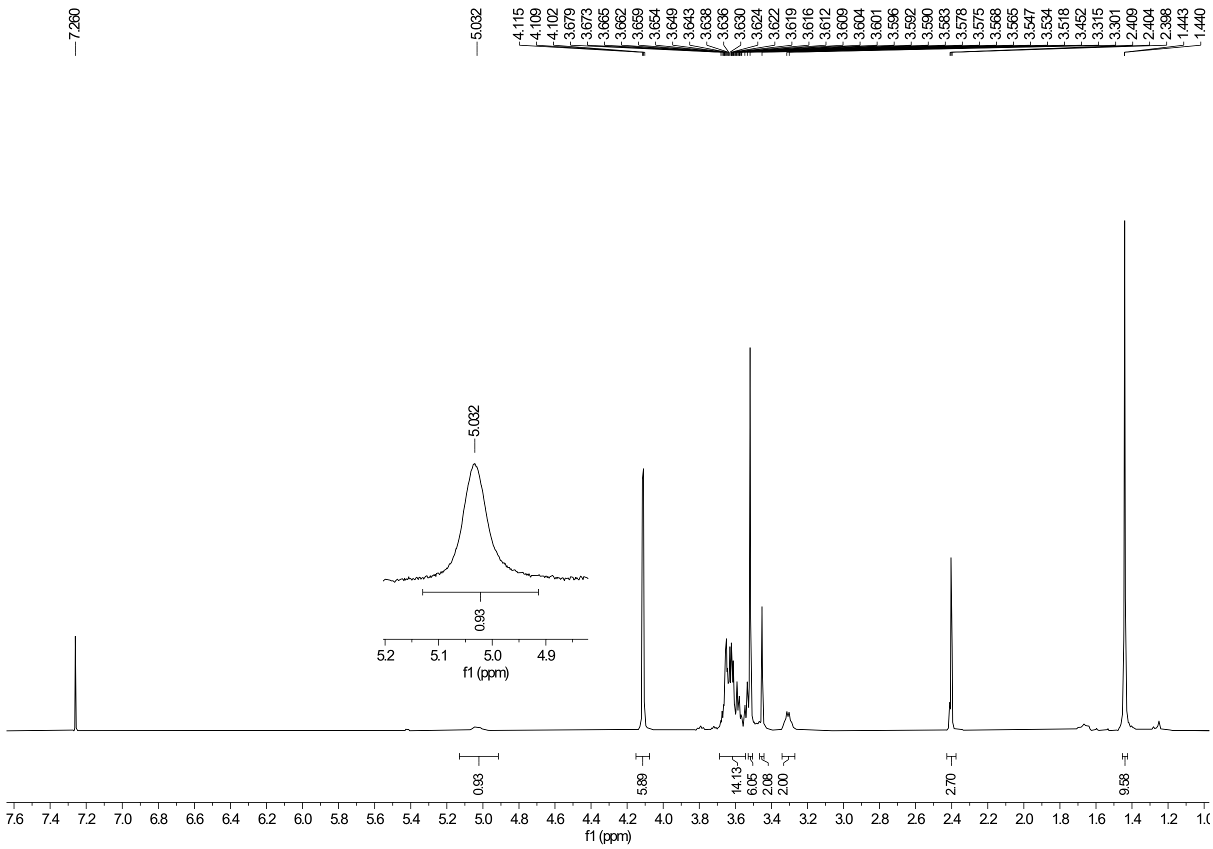

Figure S8: ^{1}H NMR spectrum of compound **3** (CD_3Cl, 400 MHz).

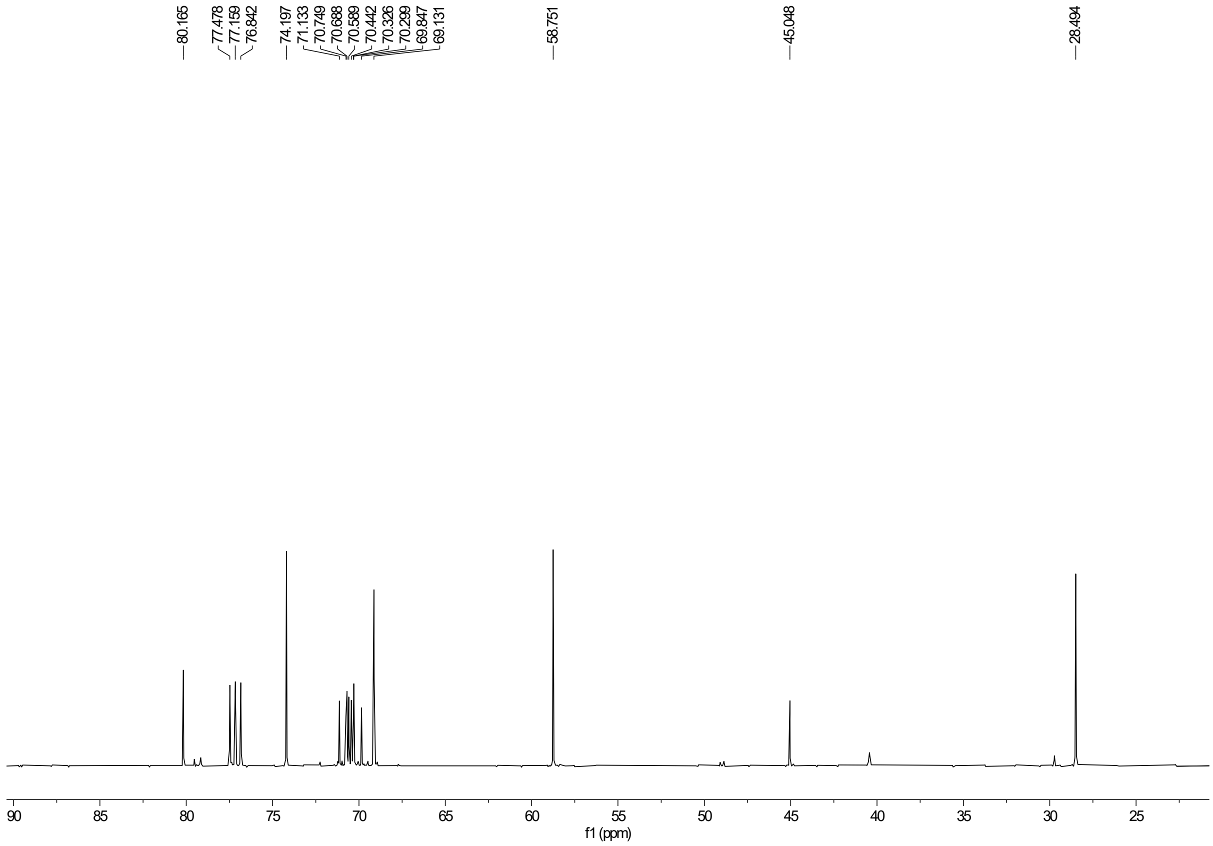

Figure S9: ^{13}C NMR spectrum of compound **3** (CD_3Cl, 100 MHz).

Lactose dendron 5

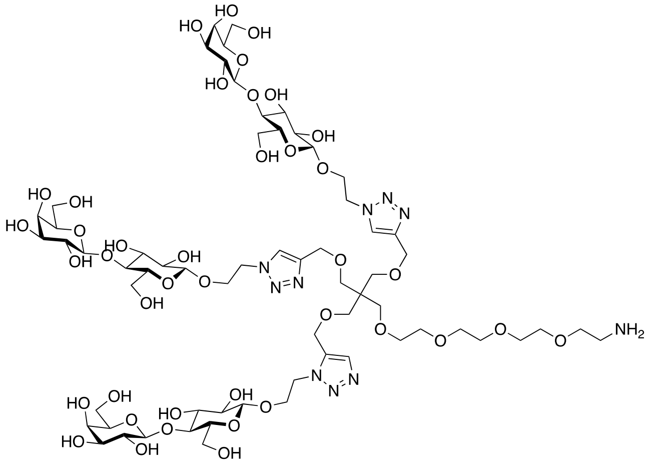

Scheme S4: Compound **5**

Compound **3** (142 mg, 0.27 mmol) was dissolved in TFA/DCM (20%, 5 mL) and the reaction was stirred at room temperature for 30 min. After that, the solvent was removed and co-evaporated several times with $CHCl_3$, yielding the unprotected derivative as a clear oil. This

derivative, 2-azidoethyl lactose **4** (400 mg, 0.97 mmol), $CuSO_4 \cdot 5H_2O$ (20.5 mg, 81.09 µmol), TBTA (89 mg, 0.16 mmol) and sodium ascorbate (42 mg, 0.24 mmol) were dissolved in H_2O/DMSO (1:1, 3 mL) in a sealed microwave vial. The solution was heated at 60 ºC in a microwave oven for 45 min. A metal scavenger resin, QuadrasilMP, was added to the reaction solution and stirred for 15 min at room temperature. After that, the mixture was filtered and the resulting solution was purified by size-exclusion chromatography (Sephadex G-25, H_2O/MeOH 9/1), furnishing the glycodendron **5** (400 mg, 89%) as a white amorphous solid. ^{1}H NMR (400 MHz, D_2O) δ 8.08 (s, 3H), 4.70 (m, 6H), 4.58 (s, 6H), 4.47 (t, J = 8.5 Hz, 6H), 4.32 (m, 3H), 4.14 (m, 3H), 3.99 – 3.92 (m, 6H), 3.83 – 3.55 (m, 41H), 3.47 (s, 6H), 3.42 (s, 2H), 3.31 (t, J = 7.6 Hz, 3H), 3.24 (t, J = 5.3 Hz, 2H); ^{13}C NMR (101 MHz, D_2O) δ 144.1, 125.7, 103.0, 102.3, 78.5, 75.4, 74.8, 74.3, 72.9, 72.6, 71.0, 70.5, 69.7, 69.6, 69.6, 69.5, 69.2, 68.6, 68.3, 68.1, 66.4, 63.6, 61.0, 60.1, 50.3, 46.7, 44.7, 39.1; ESI-HRMS for $C_{64}H_{111}N_{10}O_{40}$ [M + H]$^+$ calcd: 1659.6959, found: 1659.6951.

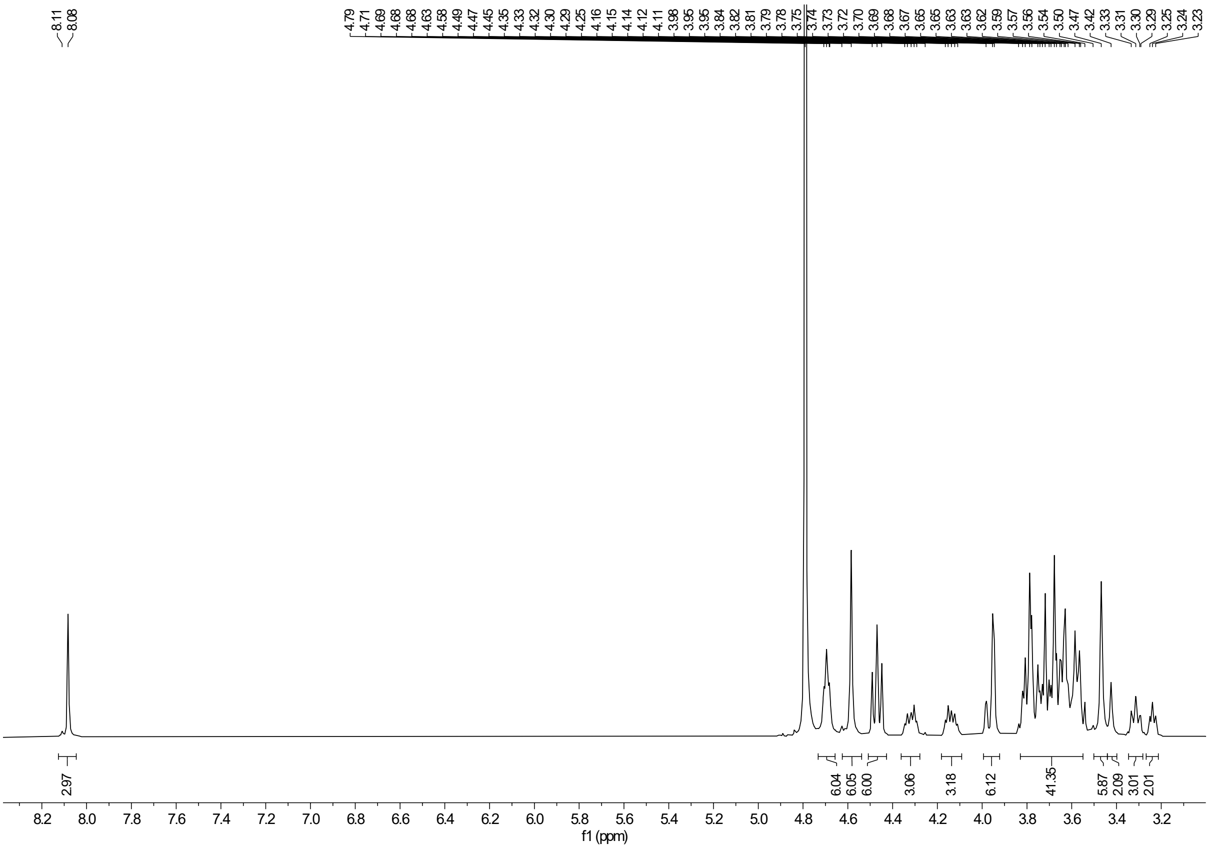

Figure S10: ^{1}H NMR spectrum of compound **5** (D_2O, 400 MHz).

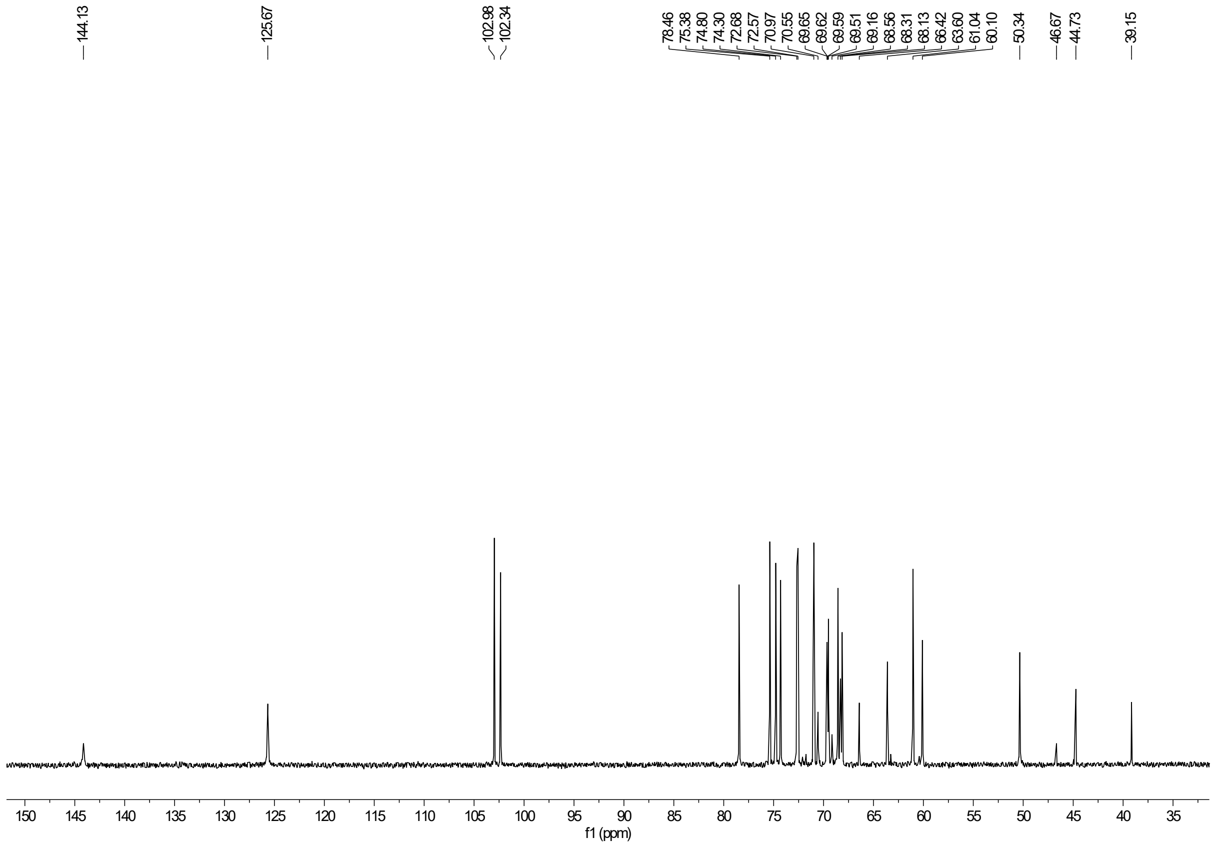

Figure S11: ^{13}C NMR spectrum of compound **5** (D_2O, 100 MHz).

4. Synthesis of lactose-CDs 7-8.

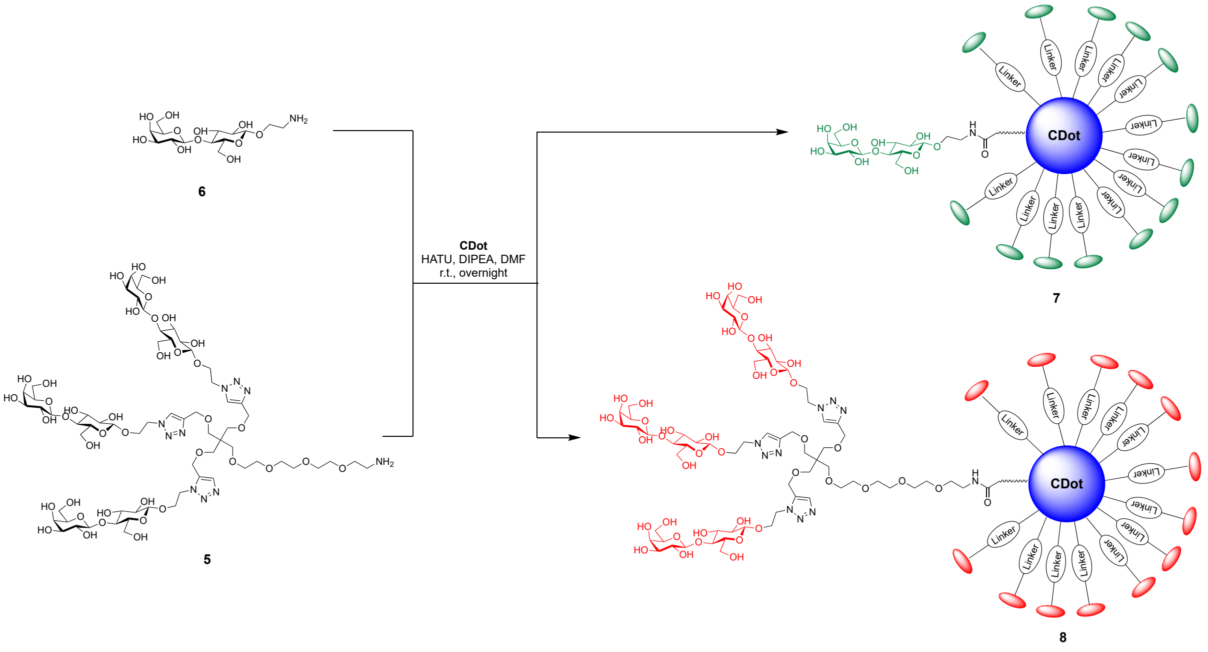

Scheme S5: The synthesis of Lactose-CDs **7** and **8**.

Lactose-CD* (CD-Lac) *7.

To a solution of 2-aminoethyl lactose **6** (40 mg) and DIPEA (21 mL) in anhydrous DMF (3 mL), a solution of CD (20 mg) and HATU (49 mg) in anhydrous DMF (3 mL) was added. The reaction mixture was stirred at room temperature overnight. After that, the solvent was removed, and the resulting crude was dissolved in 0.1M aq. NaOH (6 mL) and stirred at room temperature for 1 h. The solution was neutralized with 0.1M aq. HCl (6 mL) and washed with Et_2O (three times). The aqueous layer was dialyzed (MWCO 500-1000) for 24 h and lyophilized to afford lactose-CD **7** (24 mg) as a brown solid.

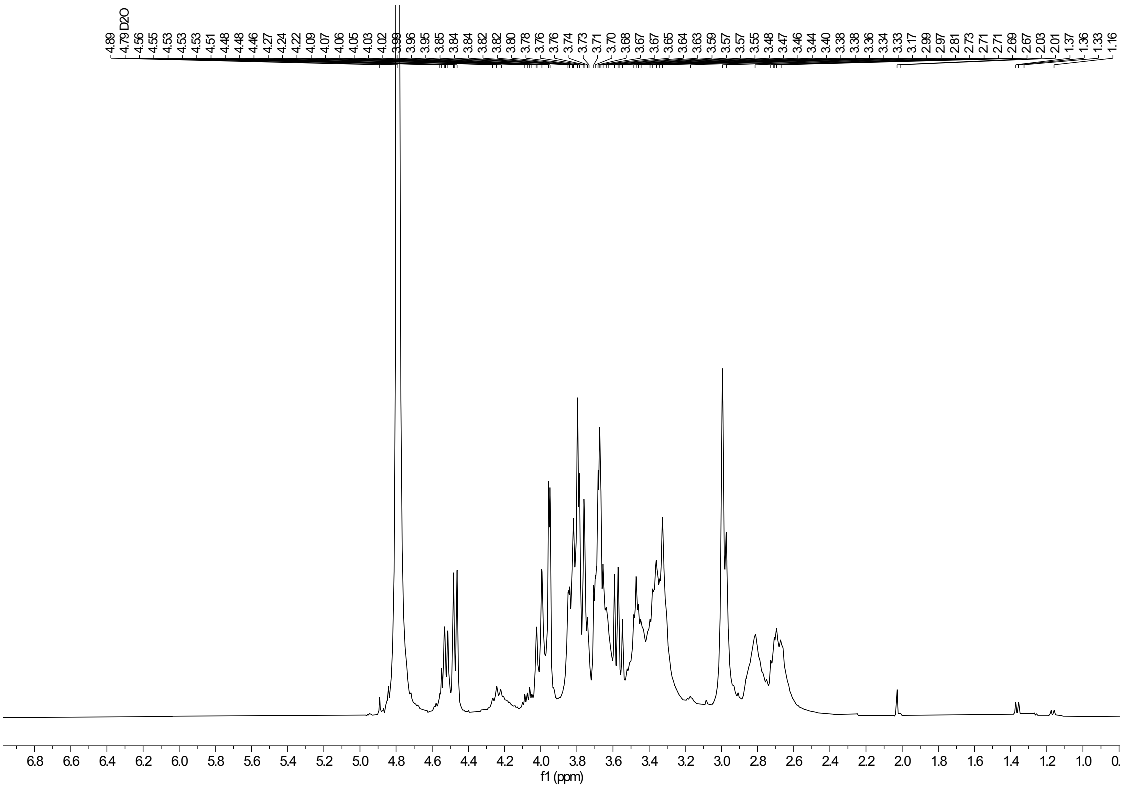

Figure S12: ^{1}H NMR (D_2O, 400 MHz) spectrum of CD-Lac **7**.

Figure S13: ^{1}H-^{13}C HSQC NMR (D_2O) spectrum of CD-Lac **7**.

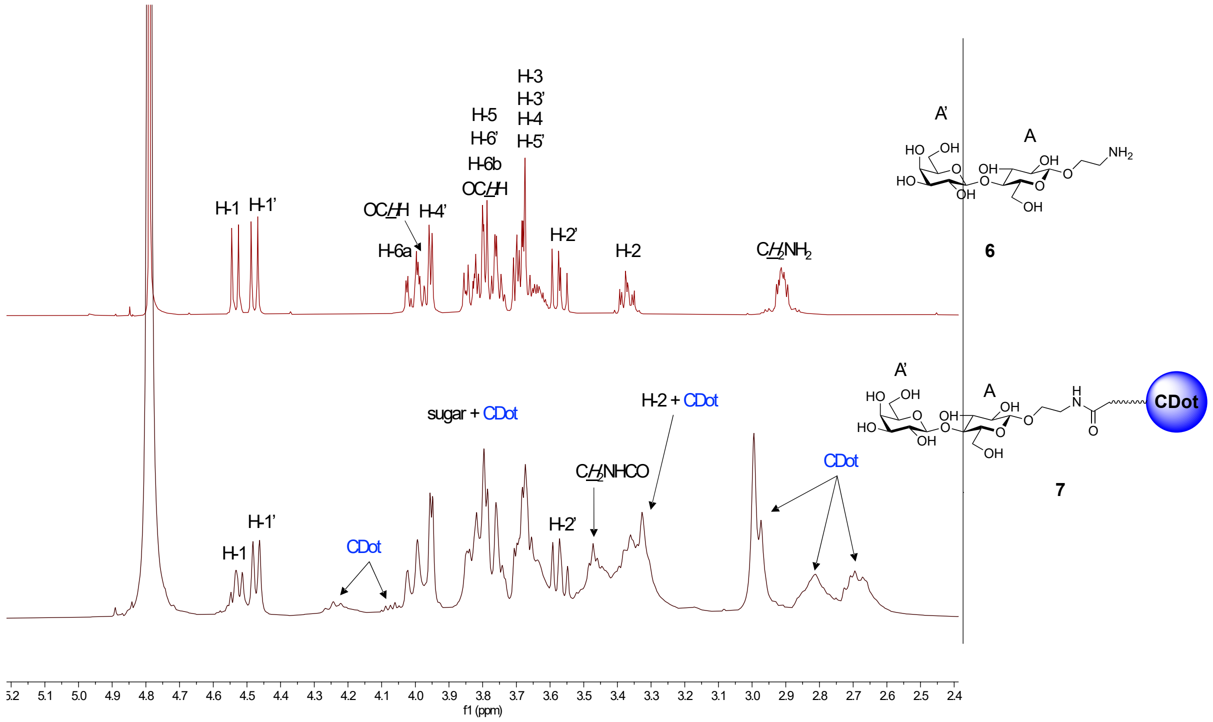

Figure S14: ^{1}H-NMR spectra of A) Lactose **6** and B) CD-Lac **7**, showing key signals (e.g. H1, H1'attributed to lactose anomeric signals and signals associated to linker CH_2NH_2 of **6** and CH_2NHCO of **7** amide bond) which demonstrates glycoside conjugation to the CD).

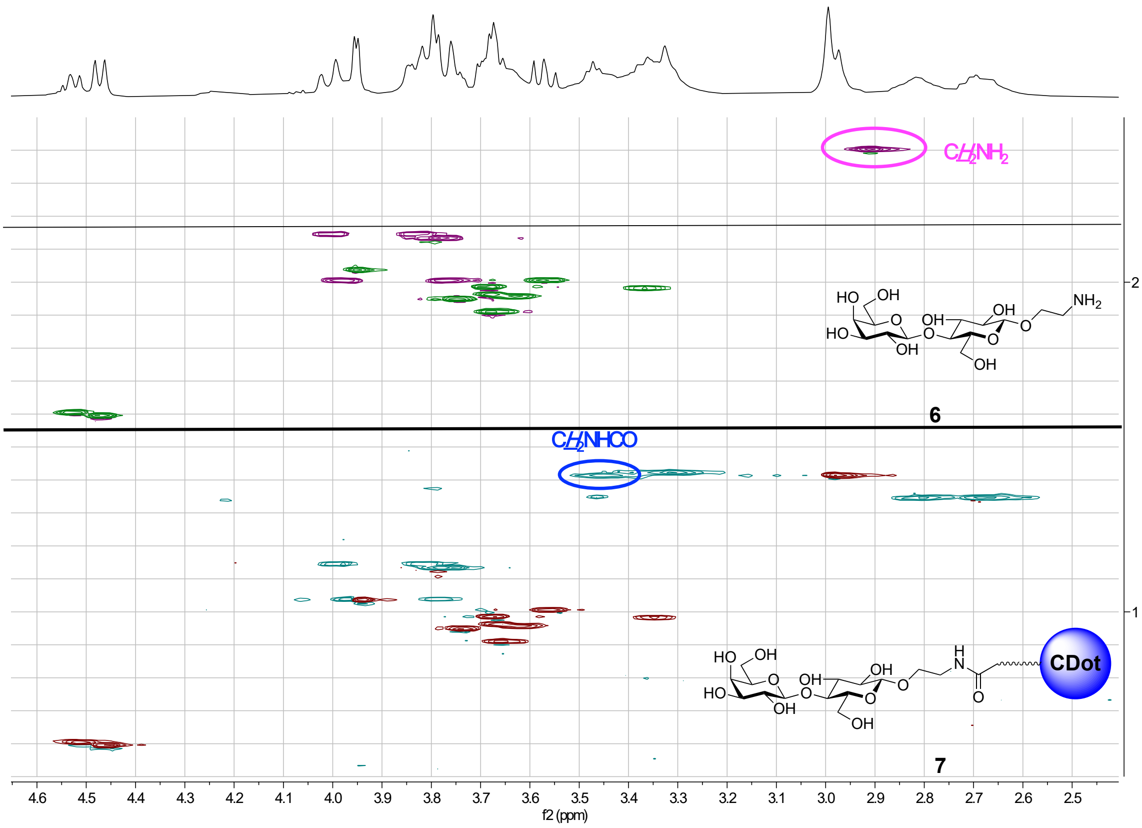

Figure S15: HSQC spectra of Lactose **6** (top) and CD-Lac **7** (bottom). Showing chemical shift of linker signals CH_2NH_2 of **6** and CH_2NHCO of **7** upon CD conjugation.

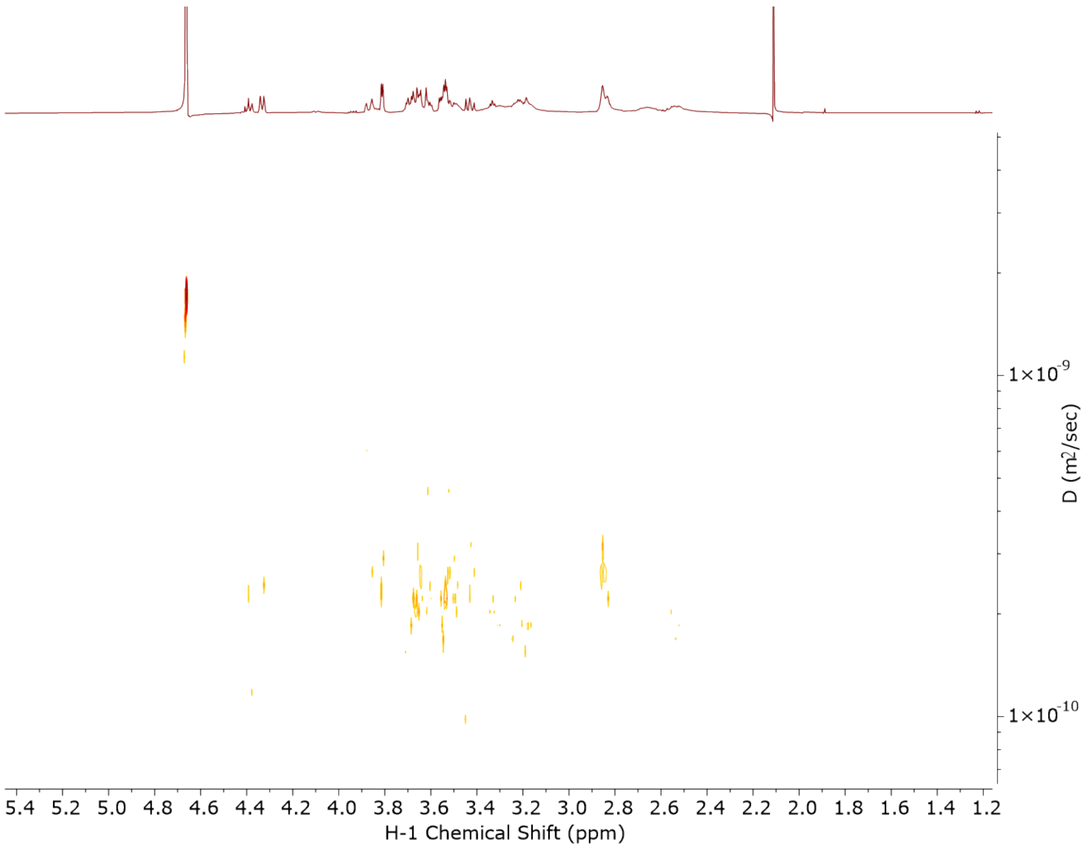

D_w (m^2 s^{-1})	D_{CD} (m^2 s^{-1})	σ $_{CD}$ (nm)
1.75 x 10^{-9}	2.16 x 10^{-10}	1.11

Figure S16: DOSY and ^{1}H NMR spectra of CD-Lac **7**.

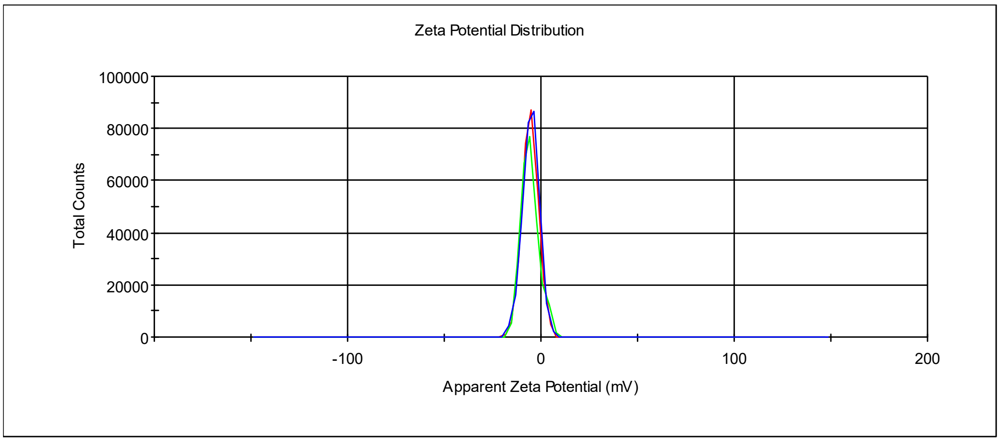

-5.48 ± 0.26 mV

Figure S17: Zeta-potential measurement of CD-Lac **7**. Each line represents individual measurements of the same sample, which were used to obtain an average value.

(Lactose)$_3$-CD* (CD-Lac3) *8.

To a solution of lactose dendron **5** (60 mg) and DIPEA (16 mL) in anhydrous DMF (3 mL), a solution of CD (15 mg) and HATU (27 mg) in anhydrous DMF (3 mL) was added. The reaction mixture was stirred at room temperature overnight. After that, the solvent was removed, and the resulting crude was dissolved in 0.1M aq. NaOH (6 mL) and stirred at room temperature for 1 h. The solution was neutralized with 0.1M aq. HCl (6 mL) and washed with Et_2O (three times). The aqueous layer was purified by ultrafiltration (Amicon® Ultra-15, MWCO 3 kDa) and lyophilized to afford (Lactose)$_3$-CD **8** (39 mg) as a brown solid.

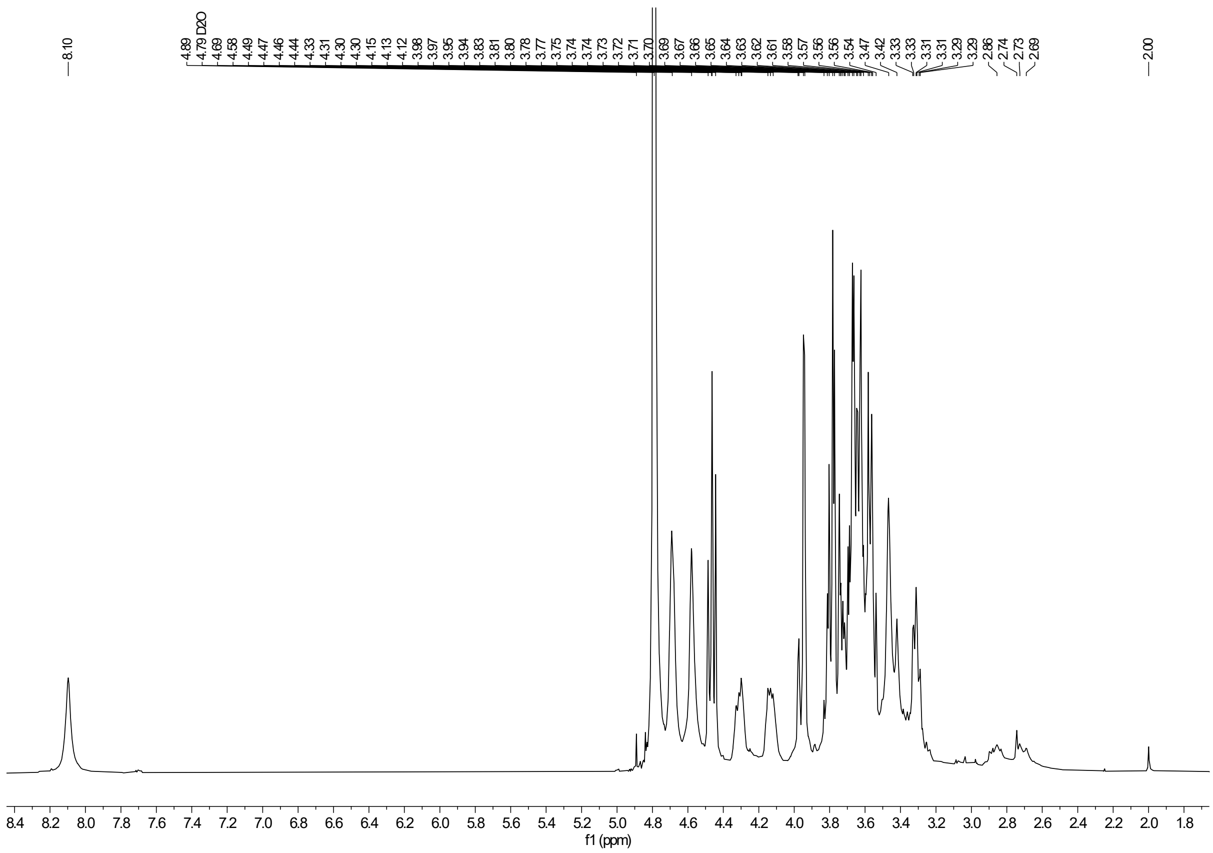

Figure S18: ^{1}H NMR (D_2O, 400 MHz) spectrum of CD-Lac3 **8**.

Figure S19: ^{1}H-^{13}C HSQC NMR (D_2O) spectrum of CD-Lac3 **8**.

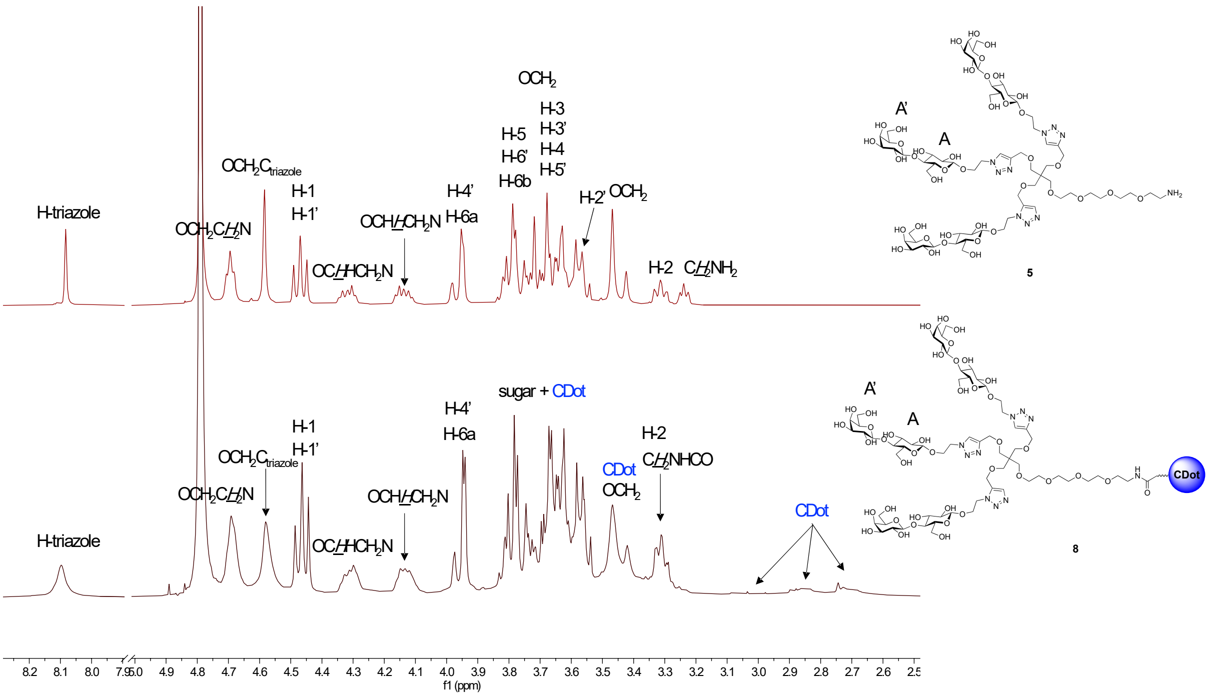

Figure S20: H-NMR spectra of A) Lactose dendron **5** and B) CD-Lac3 **8**, showing key signals (e.g. H1, H1'attributed to lactose anomeric signals and signals associated to linker CH_2NH_2 of **5** and CH_2NHCO of **8** amide bond) which demonstrates glycoside conjugation to the CD).

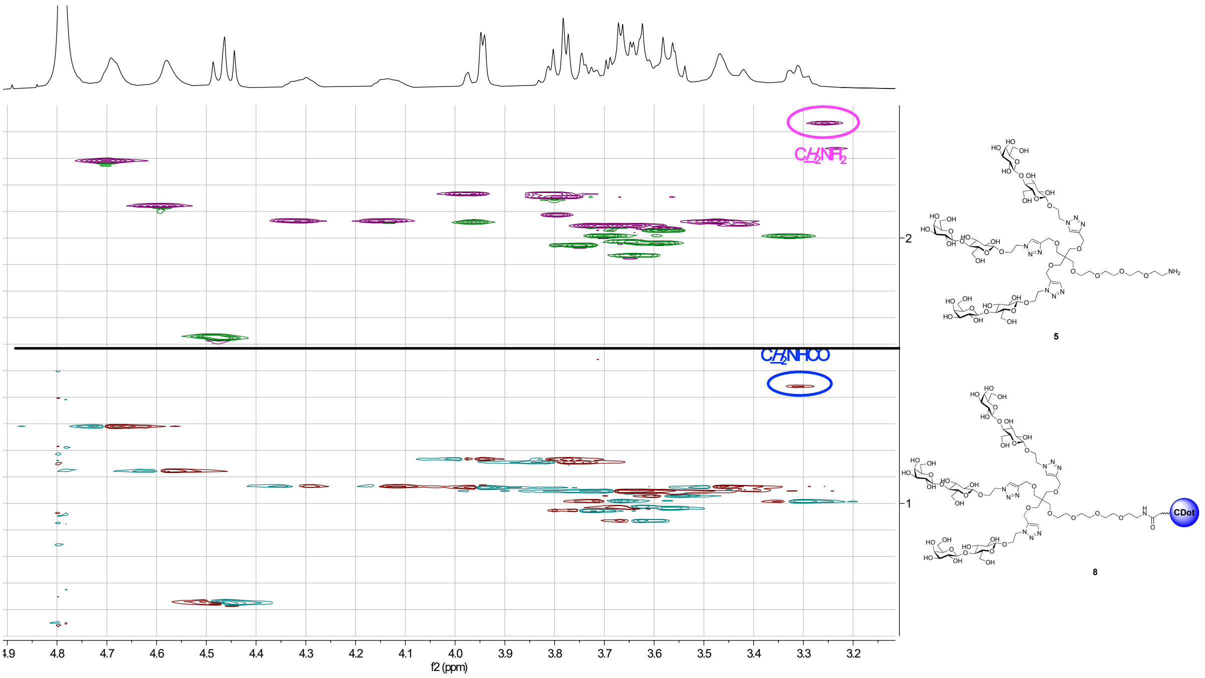

Figure S21: HSQC spectra of Lactose dendron **5** (top) and CD-Lac3 **8** (bottom). Showing chemical shift of linker signals CH_2NH_2 of **5** and CH_2NHCO of **8** upon CD conjugation.

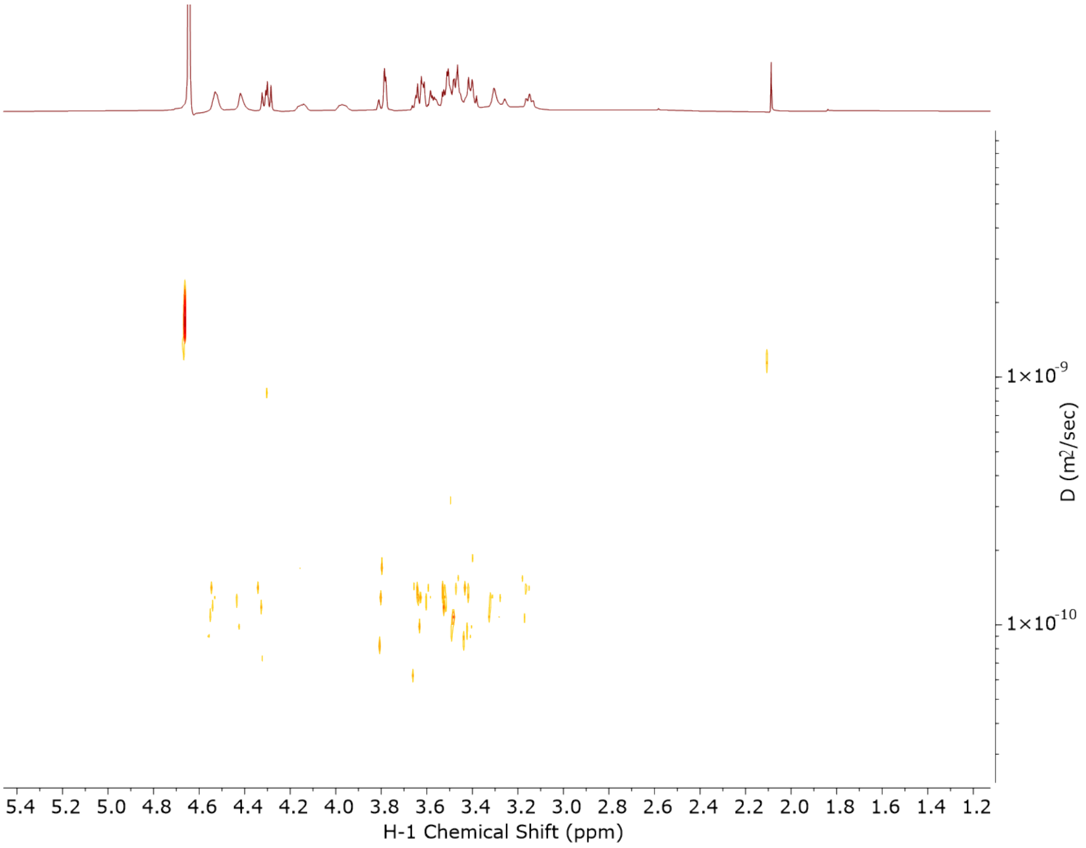

D_w (m^2 s^{-1})	D_{CD} (m^2 s^{-1})	σ $_{CD}$ (nm)
1.71 x 10^{-9}	1.24 x 10^{-10}	1.9

Figure S22: DOSY and ^{1}H NMR spectra of CD-Lac3 **8**.

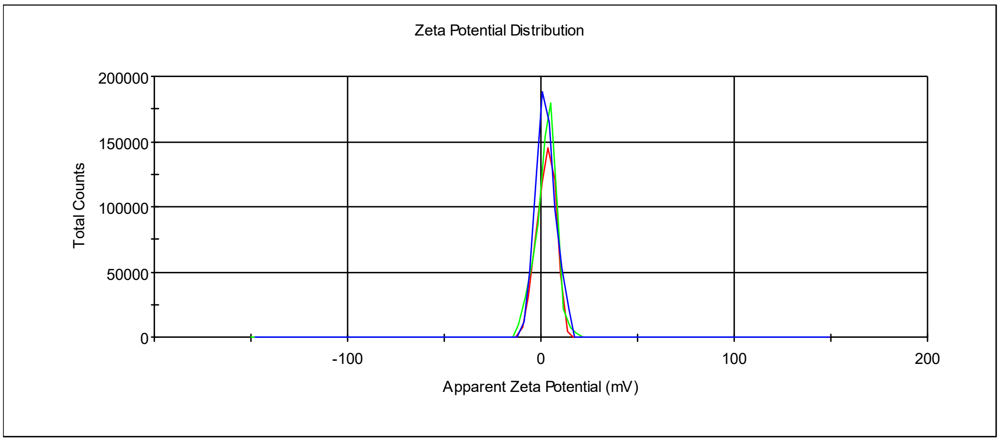

2.67 ± 0.08 mV

Figure S23: Zeta-potential measurement of CD-Lac3 **8**. Each line represents individual measurements of the same sample, which were used to obtain an average value.

References

[1] M. Ortega-Muñoz, J. Lopez-Jaramillo, F. Hernandez-Mateo and F. Santoyo-Gonzalez, *Adv. Synth. Cat.*, **2006**, *348*, 2410-2420.

[2] K. S. Yang, G. Budin, C. Tassa, O. Kister and R. Weissleder, *Angew. Chem. Int. Ed.,* **2013**, *52*, 10593-10597.

[3] Y. Koshi, E. Nakata, M. Miyagawa, S. Tsukiji, T. Ogawa and I. Hamachi, *J. Am. Chem. Soc.*, **2008**, *130*, 245-251.

[4] G. Canzi, A. A Mrse, and C.P. Kubiak. *J. Phys Chem C*, **2011**, 115(16), 7972–7978.

[5] S. Mondal, A. Yucknovsky, K. Akulov, N. Ghorai, T. Schwartz, H. N. Ghosh, N. Amdursky, *J Am Chem Soc* **2019**, *141*, 15413-15422.

IV - Atomic Force Microscopy Data

Samples for atomic force microscopy were prepared through drop casting of 5µl, 1 mg/ml CDs onto freshly cleaved mica. A 10 second interval was allowed for particles to settle to the surface before the droplet was removed with a flow of compressed nitrogen.

Atomic Force Microscopy imaging was performed utlising a Multi-mode VIII microscope with Nanoscope V controller combined with a Fastscan head unit (Bruker, CA, USA). PeakForce feedback control was implemented to ensure that imaging remained in a sub 1 nN force regime. To resolve CDs SCANASYST-AIR-HR cantilevers were used with nominal tip radius and spring constant of 2 nm and 0.4 N/m respectively.

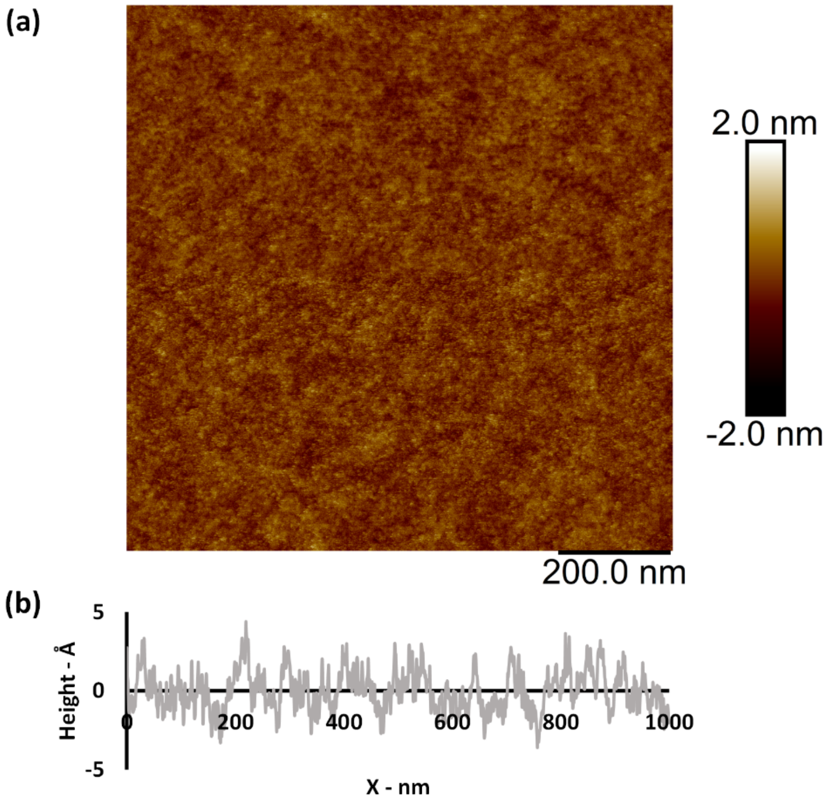

Figure S24: a) A topographic map of the surface of mica. **b)** A line profile across (a) showing the roughness of the background surface in AFM experiments.

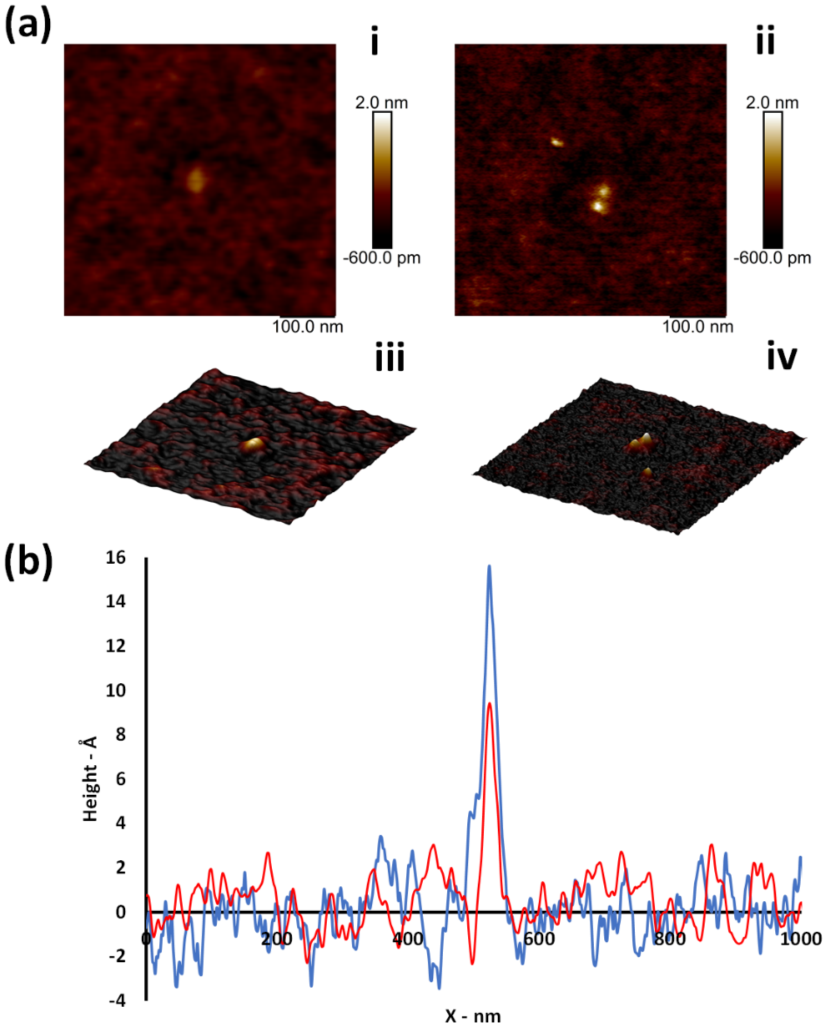

Figure S25: a) Topographic map and 3-dimensional surface render of carbon dots CD **(i, iii)** and of functionalised carbon dots CD-Lac3. **b)** A line profile across examples of CD (Red) and CD-Lac3 (Blue). Note that the roughness of the background substrate is equivalent to that of the mica displayed in Figure S23 indicating that there is little impurity or residues in the samples.

V - Medium Angle X-Ray Scattering

Experimental

Samples were prepared by pipetting 70μL of solution into a 1.5 mm borosilicate glass capillary (Capillary Tube Supplies Ltd) and sealed using UV curable adhesive (Norland) for 30 minutes under UV light.

SAXS experiments were performed on a Ganesha 300XL scattering instrument (Xenocs) over a Q range $0.015 < Q < 0.65$ Å^{-1} an exposure time of 3600 s. Data were corrected for

transmission and absolute intensity and reduced using SAXSGUI. A solvent background was collected and subtracted from each data set. Data were fitted to a spherical form factor using SASView 4.0 (www.sasview.org).

Results

Sample	CD	CD Lac	CD Lac 3
Scale	0.00095 ± 4.6 x 10^{-5}	0.0011 ± 3.2 x 10^{-5}	0.00075 ± 1.3 x 10^{-5}
Background	0.0057 ± 7.0 x 10^{-5}	0.0084 ± 6.4 x 10^{-5}	0.0038 ± 4.9 x 10^{-5}
Radius / Å	9.81 ± 0.18	11.51 ± 0.13	15.95 ± 0.12
Chi^2	1.41	1.89	2.26

Table S3: Fitting parameters for solution SAXS of bare and functionalised carbon dots fitted to a spherical form factor model.

The SAXS data for each of the samples shown in Fig. B are not asymptotic at low Q, indicating that there are larger scattering objects in solution and that the samples are polydisperse. This can be observed also in the mismatch between the model fit and the experimental data at low Q. A Schulz distribution was added to the form factor model to take into account polydispersity, however this did not return a physically relevant fit. There is low contrast between solvent (water) and sample (primarily carbon-based materials), as such the scattering intensity is low for all samples.

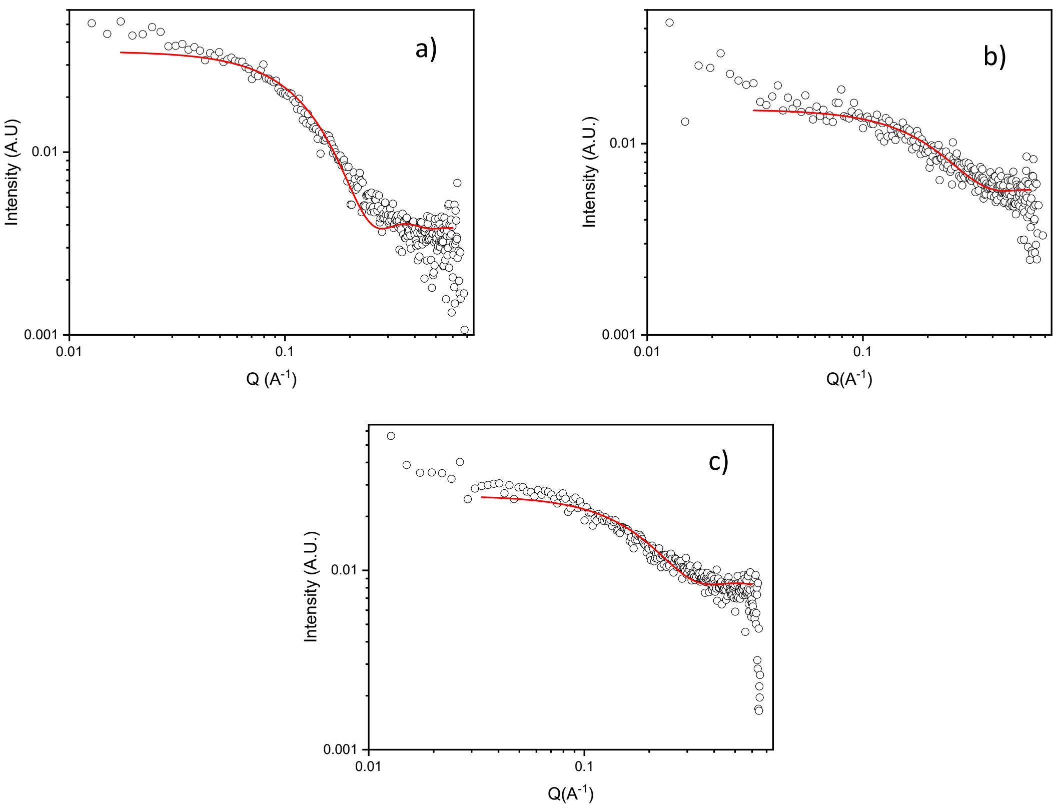

Figure S26: Small Angle X-ray Scattering Patterns of **a)** CD Lac3, **b)** CD, **c)** CD Lac.
